# Detection of Arsenic at Micromolar Concentrations and Remediation of Arsenic from Drinking Water with a Bemliese Teabag

**DOI:** 10.1021/acsomega.5c12885

**Published:** 2026-03-19

**Authors:** Vick J. Tan, Manuel A. Lema, Keidy L. Matos, Adam B. Braunschweig

**Affiliations:** a Advanced Science Research Center, Graduate Center, 449003 City University of New York, 85 St. Nicholas Terrace, New York, New York 10031, United States; b EP Academy, 12310 Singletree Lane, Eden Prairie, Minnesota 55344, United States; c Scarsdale High School, 1057 Post Road, Scarsdale, New York 10583, United States; d Department of Chemistry, Hunter College, 695 Park Avenue, New York, New York 10065, United States; e PhD Program in Chemistry, Graduate Center, City University of New York, 365 fifth Avenue, New York, New York 10016, United States

## Abstract

Arsenic (As) contamination in groundwater is a global public health concern, with many water sources across the globe exceeding the World Health Organization’s maximum permissible limit of 10 μg·L^–1^. Health issues related to As in groundwater are most prominent in South and Southeast Asia, where approximately 180 million people are exposed to dangerous As levels. To address this global health challenge, there exists a need for new, cost-effective methods to detect and remove As in water. Measurements of As levels in water are most typically performed in the field using colorimetric test strips, which are hard to use and not widely accessible. To address these ongoing detection challenges, we developed an accurate and affordable assay for the detection of As that involves the dissociation of NaAsO_2_ in the presence of HCl and KIO_3_ to form AsI_3_, which has a strong yellow color. Current methods to remove As from water mainly focus on wastewater treatment by reverse osmosis, which is expensive and not available to all communities vulnerable to As contamination. Here, Bemliese teabags embedded with magnetic iron oxide nanoparticles (MIO-NPs) and filled with 5 g of pulverized eggshells are shown to be a cost-effective and accessible way to remove >98% of As from a 50 mL solution initially containing 35 mg·L^–1^ NaAsO_2_ after 6 h of contact at near-neutral pH (∼7), reducing [As] to 0.69 mg·L^–1^. Taken together, the combination of new methods for As detection and removal provides scalable solutions to the global health challenges posed by As contamination in drinking water.

## Graphical abstract

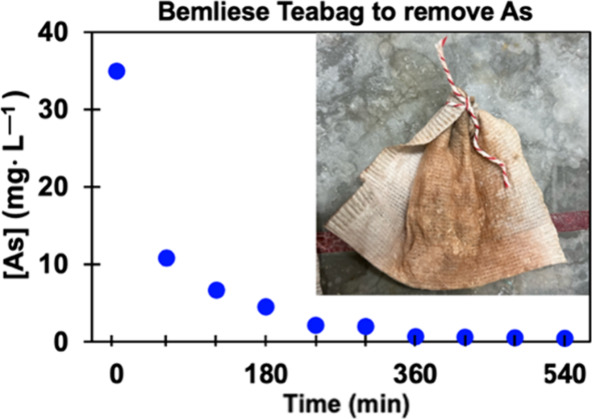

## Introduction

Arsenic (As) concentrations of 5 mg·L^–1^, 500x greater than the World Health Organization (WHO) maximum permissible limit of 10 μg·L^–1^, are common near anthropogenic sources of As.^1^ The two predominant inorganic oxidation states of As in water, arsenite (As­(III)) and arsenate (As­(V)), are carcinogens associated with human skin, lung, bladder, kidney, and liver cancers, cardiovascular diseases, stunted growth, and developmental problems such as autism^1–3^ in children, with As­(III) generally regarded as more toxic and more mobile than As­(V). Even low concentrations of As pose a risk if exposure continues over long periods,^4^ underscoring the need for effective monitoring and remediation techniques. Contamination from mining and fracking, coal-fired power plants, erosion runoff from mountains, As-treated lumber, and As-containing pesticides contribute to high levels of As in drinking water.^5^ In some tube wells in Bangladesh, for example, As concentrations as high as 4.7 mg·L^–1^ have been detected.^6^ As is found in concentrations dangerously above the WHO limits in other densely populated regions in South and Southeast Asia, including Bihar, India, Dhaka, Bangladesh, and Hanoi, Vietnam (Figure 1A). These cities all have a higher prevalence of children with stunted growth and autism (Figure 1B), both of which are known symptoms of As toxicity.^3,7^ Extensive studies in the Antofagasta region of Chile, which once had As levels as high as 600 μg·L^–1^, correlated As overexposure to increased rates of lung and bladder cancer mortality for men and women.^8^ Remediation efforts in Antofagasta have lowered As levels to below 100 μg·L^–1^, and since then, falling lung cancer mortality rates (Figure 1C) have been correlated to the decreased [As] in drinking water.

**Figure 1:**
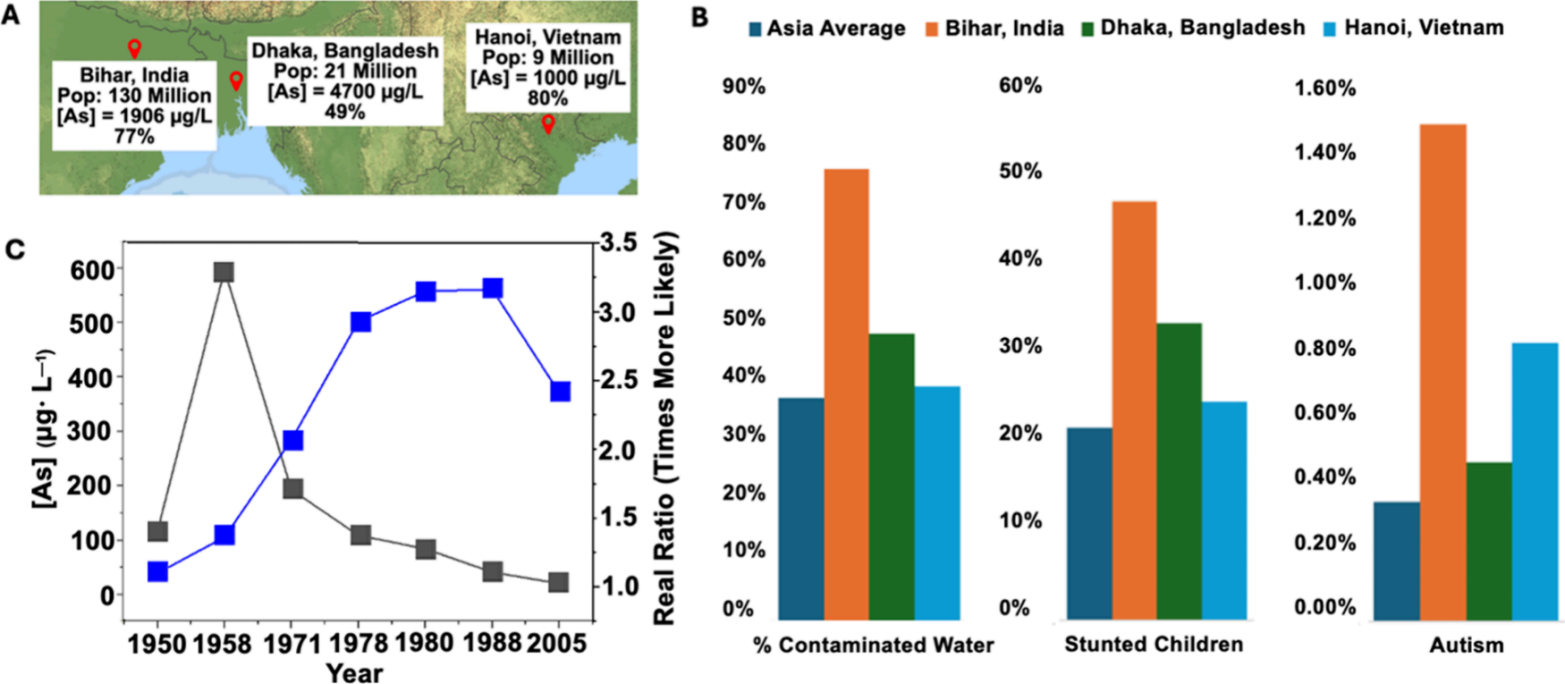
(A) Three cities with high [As] in drinking water (location; population; highest [As]; percent of water source above the WHO’s permissible limit). (c) StepMap, 123map, data: OpenStreetMap, License ODbL 1.0. (B) Left; percent of water contaminated with As over the WHO’s permissible limit plotted with known consequences of As contamination. Center; stunted growth; right; autism in Asia, Bihar, Dhaka, and Hanoi. Percentages for As over the WHO’s permissible limit are 38, 77, 49, and 40% for the Asia Average, Bihar, Dhaka, and Hanoi, respectively.^9–12^ Percentages for stunted growth are 22, 48, 34, and 25% for the Asia Average, Bihar, Dhaka, and Hanoi, respectively.^13–16^ Percentages for autism are 0.36, 1.50, 0.48, and 0.84% for the Asia Average, Bihar, Dhaka, and Hanoi, respectively.^17–20^ (C) Lung cancer mortality rates (blue) for men 30 years and older plotted with [As] levels (black) in the Antofagasta region, Chile.^8^

The challenge of analyzing drinking water samples for As contamination is significant because of the manpower and instrumentation requirements, making the detection of As difficult in areas where it is most needed.^21^ Analytical methods capable of detecting As are usually based on expensive and complex laboratory instrumentation, such as atomic absorption spectroscopy, induced coupled plasma atomic emission spectroscopy, X-ray fluorescence, and atomic fluorescence spectroscopy, and As salts can often damage the equipment.^21^ As such, these laboratory methods are unsuitable for the high-frequency, low-cost, and on-site As monitoring needed by many communities affected by As contamination in their drinking water. Analysis using optical detection systems minimizes fouling effects by avoiding the need for direct contact between the sample and the sensor, and colorimetric tests can also be analyzed by eye, resulting in a simple and low-cost detection solution, making them suitable for on-site or at-home heavy metal monitoring in resource-limited situations.^21^ Thus, an ideal practical screening method for many affected settings is a low-cost colorimetric approach that is widely available and quantitatively useful across contaminated water concentrations. However, quantitative confirmation at or below the WHO guideline level (10 μg·L^–1^)^45^ generally requires advanced atomic spectroscopic techniques (e.g., ICP-MS), and therefore UV–vis colorimetric assays are best positioned for screening and quantification in contaminated regions rather than compliance verification at trace levels.^22,23^

In addition to detection challenges, there also exist significant problems with current As removal strategies because they cannot be deployed where they are most needed. Typically, As is removed from drinking water by reverse osmosis, which is expensive, involves significant infrastructure, and discards 70–80% of the water.^22^ Furthermore, As mitigation strategies focus on removal from wastewater,^23^ while As poisoning is most often caused by drinking water.^1^ Thus, there is still a need for methods for removing inorganic As from water that are affordable and widely accessible and are a solution specifically for As-related drinking water challenges. In recent years, iron oxide nanoparticles (MIO-NPs) have received attention in biomedical and healthcare applications because of their magnetism, low toxicity, biocompatibility, and biodegradability.^24^ MIO-NPs have been shown previously^25,49,50^ to remove heavy metals from water. For example, polyvinylpyrrolidone-coated MIO-NPs remove Cd^2+^, Cr^6+^, Ni^2+^, and Pb^2+^ from synthetic soft water and seawater.^25^ 167 mg·L^–1^ of MIO-NPs could remove nearly 100% of the four metals^25^ at 0.1 mg·L^–1^ and more than 80% at 1 mg·L^–1^. However, while MIO-NPs may remove heavy metals from H_2_O, they require a strong magnet to remove the nanoparticles,^25^ which is not a widely accessible, scalable, and cost-effective solution to the As removal challenge. MIO-NPs have also been used to remove As in the form of nanowire mats, which operate similarly to a filter.^26^ Additionally, it has been shown recently that the brewing of tea itself has metal-remediating effects^27^ as a result of absorption of the metal onto the teabag fibers. However, MIO-NPs have not yet been embedded into teabags themselves, which could combine the benefits of MIO-NPs and the ability of teabags to absorb metals, resulting in an affordable strategy for removing large quantities (>5 mg·L^–1^) of As contamination from water.

Combining the high absorbance of Bemliese fabric with the As-binding potential of eggshells and MIO-NPs offers a promising strategy for efficient and cost-effective As remediation in water. Bemliese fabric, which is a cellulose-based, continuous, nonwoven fabric,^28^ has recently been used to make teabags, and is noteworthy in that it has extremely high liquid absorption and retention capabilities, which is important for the flow of As-contaminated water through the teabag. Eggshell-derived biochars have also drawn attention for water remediation, as at a pH of 4.5, biochar consisting of eggshells can remove 96% of As when [NaAsO_2_] is <0.6 mg·L^–1^ at a sorbent dose of eggshell of 0.9 g·L^–1^.^29^ Although the process of utilizing an eggshell biochar works effectively in wastewater applications, eggshell-based As removal strategies are difficult to use in drinking water, as a strong acid must be added to the water to lower the pH to the level necessary for efficient metal separation. Eggshell biochar is also difficult to contain and is not usable for the treatment of drinking water because the biochar forms a sludge at the bottom of the water column. As such, the addition of an eggshell-derived char inside of an MIO-NP embedded Bemliese fabric, where the biochar is well-contained, could serve as an ideal remediation technique for As remediation.

Here, we address the challenges of affordable detection and removal of As from contaminated drinking water. We describe a novel As detection assay—the arsenic tri-iodide assay (ATIA)—which is based on the dissociation of sodium meta-arsenite (NaAsO_2_), in which As is present in the trivalent oxidation state (As­(III)), in the presence of HCl and KI, forming AsI_3_, a compound with a distinct yellow color that enables visual observation of As contamination in water. NaAsO_2_ was selected as the model contaminant because it is the most common As species in groundwater.^44,48^ The ATIA provides reliable and quantifiable colorimetric results for As levels ranging from 10 μg·L^–1^ to 5 mg·L^–1^ using widely accessible reagents. Additionally, we introduce a teabag-based remediation approach, where MIO-NP-embedded Bemliese teabags filled with pulverized eggshells successfully remove up to 98% of As from 50 mL of a 35 mg·L^–1^ As solution. Unlike traditional detection methods, which rely on expensive and complex instrumentation, the ATIA is simple, transportable, and uses only widely available resources, making it ideal for low-income and rural communities. Similarly, the doped teabags are a sustainable and inexpensive method for reducing [As] in water below the WHO’s permissible limit and have significant benefits over reverse osmosis, which is costly, results in significant water waste, and is not a solution for drinking water sourced from local wells, which are still common in many of the afflicted areas.^22^ As such, we demonstrate scalable and practical solutions for monitoring and mitigating As contamination in drinking water, particularly for communities with limited access to advanced water treatment technologies.

## Materials and Methods

MIO-NPs were synthesized via coprecipitation of FeCl_2_ and FeCl_3_ under an inert argon atmosphere, followed by ammonium hydroxide addition and stabilization with polyvinylpyrrolidone (PVP), following reported protocols.^26^ Eggshells were prepared from locally sourced shells, cleaned, dried, mechanically milled using a ball mill, and either uncharred or charred (150 °C, ambient atmosphere, 30 min). Bemliese cellulose teabags^31^ were embedded with MIO-NPs by adding 25 g of MIO-NPs to 450 mL of DI H_2_O and 50 mL of NH_4_OH solution at 28% w/v in a 1 L round-bottom flask. After 1 h, the teabags were removed from the flask and washed in DI H_2_O to remove unbound MIO-NPs, dried, and filled with 5 g of ground eggshell before sealing with a cotton string. Absorption experiments were conducted using dilutions from metal stock solutions prepared in DI H_2_O and diluted to the desired concentrations. As quantification was performed using UV–visible spectroscopy (Shimadzu UV-1800) in quartz cuvettes. Structural and compositional characterization was conducted using SEM-EDX (FEI Helios Nanolab 660) and ATR-FTIR spectroscopy (Thermo Scientific Nicolet Summit X).

## Results and Discussion

### Arsenic Tri-Iodide Assay (ATIA) Development

The Leucomalachite Green assay has been used widely for colorimetric detection of As since 1987 ^30^ and has been optimized by Lace et al.,^21^ who outline a method to detect As in water by the liberation of I_2_. When the assay solution is exposed to As, which subsequently oxidizes leucomalachite green to malachite green, it forms a green color that is easily observed by the eye. Here, we first study this optimized Leucomalachite Green assay method^21^ to compare its performance to the ATIA under similar laboratory conditions. In the presence of NaOAc buffer, a strong green color (440–460 nm) is formed that is dependent upon [NaAsO_2_] (Figure 2). The plot of absorbance vs concentration at λ = 443 nm, which is used to quantify the [As] in samples,^21^ does not fit well to a linear regression (*R* ^2^ = 0.66) over the examined concentration range of 0.64–260 μg·L^–1^ NaAsO_2_. The deviation from linearity occurs because of overlapping bands in this spectral region from various absorbing components, which may, in part, arise from the premature oxidation of Leucomalachite Green from dissolved oxygen.

**Figure 2:**
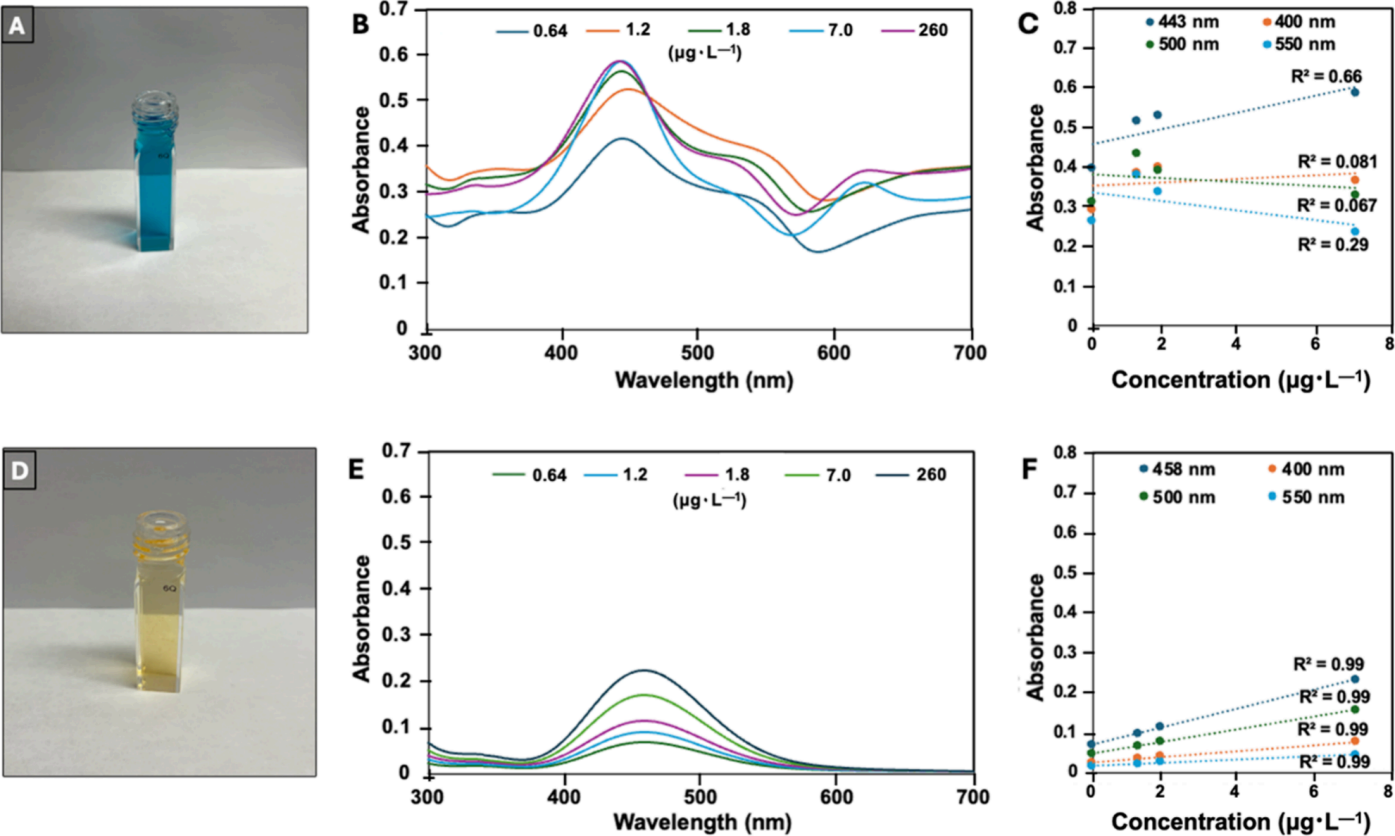
(A) Optimized Leucomalachite Green assay with 10 mg·L^–1^ NaAsO_2_. (B) UV–vis absorption spectrum of the optimized leucomalachite green assay at varying concentrations of NaAsO_2_. (C) Absorbance of the optimized Leucomalachite Green assay at varying [As] plotted at λ = 400, 443, 500, and 550 nm. (D) UV–vis absorption spectrum of ATIA for a 10 mg·L^–1^ solution of NaAsO_2_. (E) UV–vis spectroscopy of ATIA at varying concentrations of NaAsO_2_. (F) Variation of [NaAsO_2_] plotted against absorbance at λ = 400, 458, 500, and 550 nm for the ATIA.

We developed colorimetric assay for As based on the dissociation reaction of NaAsO_2_ with potassium iodate (KIO_3_) and hydrochloric acid (HCl) to form AsI_3_, a compound with a distinct yellow color (Scheme 1). We observed that adding KIO_3_ and HCl to As solutions produced a strong yellow color with a peak at λ_max_ = 458 nm, and this color change occurred without the need for Leucomalachite Green dye or NaOAc buffer. Upon exploring further, it was found that the ratio of reactants that developed the strongest color was 6 NaAsO_2_: 0.5 KIO_3_: 0.25 1 M HCl, which fully reacted with all of the NaAsO_2_ in the solution. To evaluate the ATIA assay, NaAsO_2_ solutions were prepared by the addition of NaAsO_2_, the main inorganic As compound found in water wells,^38^ to DI H_2_O at concentrations of 0.64–260 μg·L^–1^. This range was selected to span environmentally relevant As levels—from trace contamination levels commonly found in groundwater to the most elevated concentrations found in sites of As contamination. To test the samples, 1% (w/v) (KIO_3_, 0.047 M, 0.50 mL, 0.0235 mmol) and 1 M (HCl, 0.25 mL, 0.250 mmol) were added to 6 mL samples of the As solutions, and the mixture was gently shaken for 2 min. The samples were analyzed using UV–vis spectroscopy from 300 to 700 nm. In all assays where As was present, a single peak was observed at λ_max_ = 458 nm (ε = 1.2 × 10^4^ L·mol^–1^·cm^–1^). The limits of detection (LOD) and quantification (LOQ) were found to be 0.21 and 0.64 mg·L^–1^, respectively, based on an analysis of variance (ANOVA) regression analysis. All measurements were performed using NaAsO_2_ standards, analyzed by UV–vis spectroscopy from 300–700 nm, and conducted in triplicate.

**Scheme 1:**
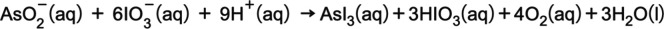
Reaction of NaAsO_2_ with I_2_ and HCl to Form the Yellow Species Arsenic Tri-Iodide (AsI_3_)

The LOD is the lowest concentration of As that can be reliably distinguished from background noise, while the LOQ is the lowest concentration that can be quantitatively measured with acceptable precision and accuracy.^31^ The LOD and LOQ present are similar to the Leucomalachite Green assay, whose LOD is 0.19 mg·L^–1^ and LOQ is 0.64 mg·L^–1^. The LOD and LOQ of the ATIA were limited by the resolution of the UV–vis spectrometer. Importantly, while the LOD and LOQ define the lowest concentrations that can be reliably distinguished from the background using UV–vis detection, the ATIA exhibits a broad linear dynamic range at higher concentrations. A linear relationship between As concentration and absorbance at 458 nm (*R* ^2^ > 0.99) is maintained from 1.4 μg·L^–1^ to 1300 mg·L^–1^, demonstrating that ATIA provides reliable quantification across concentration ranges relevant to As-contaminated groundwater systems. The full calibration range is shown in the Supporting Information (Figure S2). As such, ATIA is not intended to quantify As at the WHO guideline^45^ level of 10 μg·L^–1^, a task that typically requires ICP-MS or similarly advanced atomic spectroscopic methods. Instead, ATIA is designed for rapid, low-cost assessment of As in contaminated waters, where concentrations are commonly orders of magnitude above the WHO guidelines, particularly in As-affected groundwater systems.

The ATIA was also tested in the presence of three of the most common heavy metal contaminants found in drinking water in Bihar^32^ chromium­(III) oxide, nickel­(II) chloride, and manganese­(II) chloride—to determine if the other heavy metal contaminants affected the accuracy of the ATIA. To do so, 3 mL of 0.05 mg·L^–1^ Cr_2_O_3_ (0.00031 mmol), 3 mL of 0.5 μg·L^–1^ NiCl_2_ (2.34 × 10^–5^ mmol), and 3 mL of 0.1 mg·L^–1^ MnCl_2_ (0.00237 mmol) were each combined with 3 mL of a 3 mg·L^–1^ (NaAsO_2_, 3 mL, 0.071 mmol) sample in a vial, forming a 6 mL solution. These values were chosen to represent environmentally found concentrations of the metals found in Asia.^41,42^ Using the ATIA at the peak wavelength of 458 nm, the absorbance of the sample spiked with Cr_2_O_3_ was 0.165, a value that would correspond to a concentration of 1.79 mg·L^–1^ As, whereas the actual [As] is 1.50 mg·L^–1^, corresponding to an approximate 19% error in the absorbance values of As. The absorbance of the sample spiked with NiCl_2_ was 0.161, a value that would correspond to a concentration of 1.74 mg·L^–1^ As, corresponding to an approximate 16% error in the absorbance values of As at 1.50 mg·L^–1^. The absorbance of the sample spiked with MnCl_2_ was 0.199, a value that would correspond to a concentration of 2.16 mg·L^–1^ of As, corresponding to an approximate 44% error in the absorbance values of As (see SI Figure S17).

The cost of ATIA makes it an attractive solution for analyzing As contamination in drinking water. We calculate a cost of 0.24 USD per test, with a potential to decrease the cost to under 1 cent per test with the reusability of vials, making the ATIA highly accessible for widespread use, particularly for relatively accurate quantification of As contamination in laboratories within resource-limited settings (see Section 2.D in the SI). As such, this affordability positions the ATIA as a practical solution for monitoring As levels at environmentally relevant ranges in drinking water. We see a cost reduction of 8 cents per test and, when reusing the vials, an 87% reduction in cost for the ATIA compared to the optimized Leucomalachite Green assay.

### Teabag for Removing As from Drinking Water

Teabags for removing As from drinking water composed of Bemliese fabric, MIO-NP embedded Bemliese fabric filled with eggshell-derived biochar, or a combination of both eggshells and MIO-NPs were prepared, and their ability to remove As from contaminated water was tested through batch adsorption experiments (Figure 3). Batch adsorption experiments are tests in which a fixed amount of adsorbent is mixed with a known volume of contaminant-containing solution under controlled conditions, allowing for the evaluation of the material’s adsorption capacity by measuring the decrease in contaminant concentration over time.^33^ First, to evaluate the effect of eggshell charring on As removal, the ability of charred and uncharred eggshells to remove As from water was compared. In 250 mL beakers, solutions containing 50 mL of deionized (DI) H_2_O and 35 mg·L^–1^ of NaAsO_2_ were prepared, and 5 or 10 g·L^–1^ of charred or uncharred eggshells were added to the beaker, stirred for 20 min, and then left for 2 h. The solutions were filtered twice to remove eggshell and analyzed by the ATIA to determine the concentration of the remaining As in the water. Uncharred eggshells were more effective than charred eggshells at As removal, as charring impacted both absorbance measurements and adsorption efficiency. Based on the absorbance from the ATIA, it was observed that 5 g·L^–1^ of uncharred eggshells was the most effective for As removal, which removed 96% of As from the water. Charring the eggshells also produces a yellow color, which is then released into the solution, thus interfering with the spectrometer reading, which renders it useless for the purpose of As quantification by the ATIA. Furthermore, keratin is a natural biosorbent for As found in eggshells, and it denatures during charring,^34^ which likely decreases the effectiveness of the charred eggshells. Using the ATIA, it was found that 5 g·L^–1^ of uncharred eggshells can remove ∼96% of NaAsO_2_ from a 50 mL solution, decreasing the concentration from 35 to 1.4 mg·L^–1^. Therefore, each gram of uncharred eggshell can remove 6.7 mg of NaAsO_2_.

**Figure 3:**
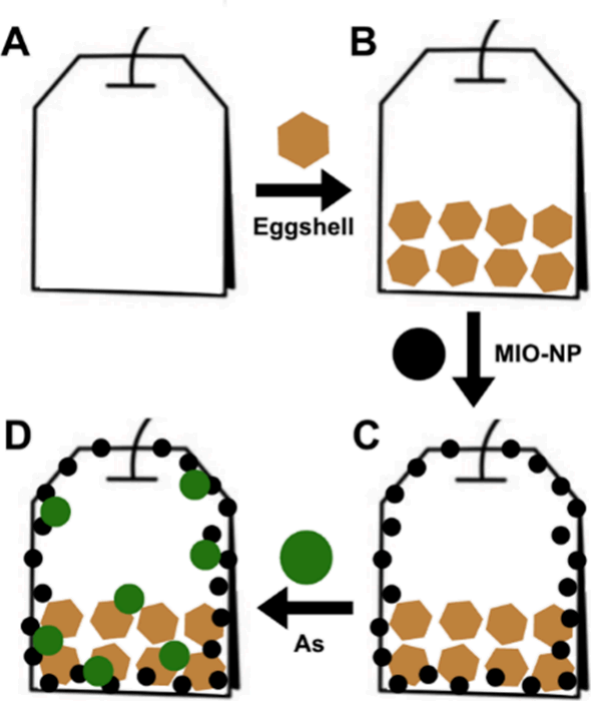
Teabag for As remediation. (A) Bemliese teabag. (B) Bemliese teabag with crushed eggshells. (C) Bemliese teabag with crushed eggshells and magnetic iron oxide nanoparticles (MIO-NPs). (D) As removal from contaminated water using a Bemliese teabag with crushed eggshells and MIO-NPs.

To explore the role of MIO-NPs on As removal, MIO-NPs were prepared as previously described^35^ and embedded into a 6 g teabag following the procedure in Kumar et al.^35^ The teabags were functionalized by immersing them in stirring or still MIO-NP solutions for 1, 3, 6, and 24 h and were characterized using energy-dispersive X-ray (EDX) analysis and scanning electron microscopy (SEM) to confirm the elemental composition and nanoparticle attachment, respectively (Figure 4). EDX confirmed the successful embedding of MIO-NP’s in the teabag fabric and the binding of NaAsO_2_ to uncharred eggshells. Fe content in the teabag sample after the MIO-NP embedding process was 9 wt %, and the elemental mapping also shows Fe on the teabag fibers, confirming the effectiveness of the embedding process. While EDX provides semiquantitative elemental analysis rather than absolute composition, this level of analysis is sufficient to confirm nanoparticle incorporation and spatial distribution on the teabag fibers. More precise bulk quantification techniques (e.g., graphene furnace atomic absorption spectroscopy) could be employed in future studies; however, such measurements are not required to support the adsorption performance trends or conclusions presented here.

**Figure 4:**
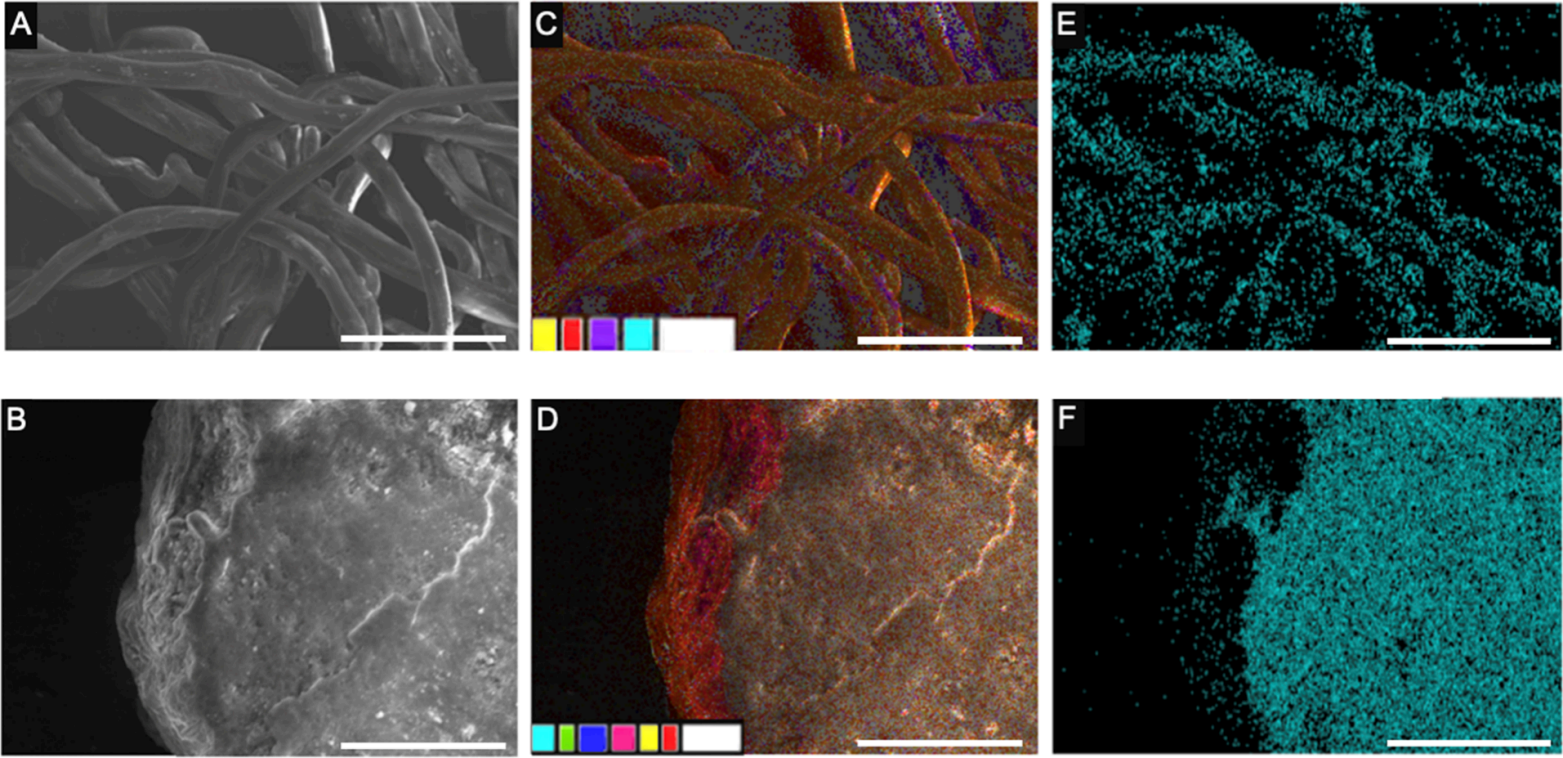
SEM and EDX analysis of a MIO-NP coated Bemliese teabag filled with eggshell after usage in As removal. (A) SEM micrograph of the teabag. (B) SEM micrograph of eggshell taken from within the teabag. (C) EDX analysis of the teabag. (D) EDX analysis of the eggshell. (E) EDX analysis with As shown on a used teabag. (F) EDX analysis with As shown on a used eggshell. The scale bar in all images is 50 μm. Colors: As – light blue, O – yellow, C – red, Fe – purple, e^–^ – white, P – green, Mg – blue, Ca – pink.

We also observe by SEM/EDX analysis that the uncharred eggshells inside the teabag bind NaAsO_2_ after the teabags are immersed in the As-spiked solution. The 2s subshells of As and Mg directly interfere with the EDX analysis, so to determine the As absorption accurately, the wt % of Mg in eggshells prior to As-incubation was determined as 0.3 wt %. After exposure to As-containing solutions, the value for Mg content as measured by EDX increased to 0.7 wt %. We attribute this 0.4 wt % increase in Mg intensity to As, and when corrected for the molecular weight of As, it leads to the determination that 0.23 wt % of the eggshells is As after immersion of the eggshell-containing teabag into the spiked solution. Furthermore, no Fe was observed on the surface of the eggshell, indicating that the MIO-NPs embedded exclusively into the Bemliese fabric and do not transfer to other surfaces.

Enthalpy (Δ*H*°) of the absorption of As onto the MIO-NP embedded teabag with the eggshell composite material was determined to understand the driving force of adsorption. Δ*H*° was calculated using the Van’t Hoff method^40^ (eq 1).

$$\ln\left(\frac{K_{f}\left(T_{2}\right)}{K_{f}\left(T_{1}\right)}\right)=-\frac{\Delta H^{{}^{\circ}}}{R}\left(\frac{1}{T_{2}}-\frac{1}{T_{1}}\right)$$ 1

where *K*_f_ is the formation constant, Δ*H*° is the standard enthalpy change (kJ·mol^–1^), *R* is the gas constant (8.314 J·mol^–1^·K^–1^), and *T* is the temperature. The *K*_f_ at each temperature was determined by measuring the equilibrium concentration of As remaining in solution after adsorption using UV–vis spectroscopy, then applying the equation *K*_f_ = *q*_e_/*C*_e_, where *q*_e_ is the mg of As absorbed per g of adsorbent at equilibrium, and *C*_e_ is the equilibrium concentration of As in solution. *K*_f_ was found to be 0.043 at room temperature, and 0.20 at 50 °C, resulting in a Δ*H*° of the MIO-NP embedded teabag and eggshells of 20 KJ·mol^–1^. From the Δ*H*° of 20 KJ·mol^–1^ and Δ*S* ° of 41.4 J·mol^–1^·K^–1^ (see SI 3.A for details), we observe that the As adsorption is endothermic, and as such, the removal method is most likely chemisorption, an entropically driven process in which adsorbate molecules form strong chemical bonds with the surface of the adsorbent. Chemisorption is typically characterized by high activation energies and is often endothermic (Δ*H*° > 0), as it requires energy input to break existing strong bonds and form new chemical interactions.^39^ In addition to the thermodynamic evidence, the observed adsorption is consistent with surface complexation mechanisms on MIO-NP and CaCO_3_-rich substrates.^46,47^ MIO-NPs provide surface hydroxyl sites capable of forming complexes with As­(III), while uncharred eggshells contribute Ca-based binding sites. The combined MIO-NP/eggshell system, therefore, enables the enhanced removal efficacy observed, relative to either component alone.

To further understand the adsorption mechanism, attenuated total reflectance Fourier transform infrared (ATR–FTIR) spectroscopy was performed on the MIO-NP-embedded Bemliese teabag and eggshell before and after As exposure (Figures S21 and S22). For the MIO-NP-embedded teabag, As adsorption leads to changes in the O–H stretching region (∼3300–3500 cm^–1^),^51^ and the appearance of new features in the ∼900–1000 cm^–1^ region^52^ is consistent with surface complexation of As­(III) on iron oxide hydroxyl sites.

Similarly, ATR–FTIR spectra of uncharred eggshells before and after As exposure show changes in the carbonate vibrational bands^53^ (∼1400–1500 cm^–1^) indicate interaction between As species and CaCO_3_ binding sites on the eggshell surface (Figure S22).

The time needed for a teabag to remove 35 mg·L^–1^ As from a solution was determined. Each teabag composition was evaluated to assess the effectiveness of As removal under varying agitation conditions and teabag compositions. The teabags were left in 50 mL of a solution containing As at 35 mg·L^–1^ for 0, 1, 3, 6, and 24 h (Table 1). The solutions were analyzed by both the ATIA and the optimized Leucomalachite Green method to determine the [As] concentration after remediation with the teabags. The results of these time trials, conducted across a range of composite teabag formulations, are summarized in Table 1.

**Table 1:** Removal of As from Water with Different Teabag Compositions[Table-fn t1fn2]

**teabag**	**time (h)**	**absorbance**	**concentration (mg L** ^**–1**^ **)**	**reduction (%)**	**As removed** **(mg L** ^**–1**^ **)**
1	0	3.2	35	N/A	0
	1	2.1	22	37	13
2	0	3.2	35	N/A	0
	1	1.7	18	51	17
3	0	3.2	35	N/A	0
	1	0.15	1.6	95	33
	3	0.19	2.0	94	33
	6	0.65	6.9	80	28
	24	0.94	12	67	23
4	0	3.2	35	N/A	0
	1	0.64	6.8	81	28
	3	0.66	7.0	80	28
	6	0.70	7.5	79	27
	24	1.0	11	69	24
5	6	0.060	0.69	98	34

a “Absorbance” refers to the UV–vis absorbance value measured during the ATIA, which quantifies the presence of tri-iodide (I_3_^–^), a product of the reaction between As and IO_3_^–^ under acidic conditions. 1: Bemliese teabags. 2: Bemliese teabags and eggshells. 3: Bemliese teabags embedded with MIO-NP’s under agitation. 4: Bemliese teabags embedded with MIO-NP’s without agitation. EDX analysis shows a increase in Fe signal intensity with increasing MIO-NP immersion time; when the Fe signal measured after 1 h of immersion is normalized to 1.0, the relative Fe signal intensities after 3, 6, and 24 h are approximately 1.3, 1.6, and 2.0, respectively. 5: Bemliese teabags embedded for 1 h without agitation combined with uncharred eggshells.

The data presented in (Table 1) reveal several important characteristics of this As remediation strategy. The Bemliese teabag alone (**1**) is able to remove 13 mg·L^–1^ of NaAsO_2_, eggshells, and the Bemliese teabag (**2**) can remove 17 mg ·L^–1^. A Bemliese teabag embedded with MIO-NPs under agitation (**3**) can remove 33 mg·L^–1^, a Bemliese teabag embedded with MIO-NPs without agitation (**4**) can remove 28 mg·L^–1^, and a Bemliese teabag embedded, combined with eggshells and without agitation (**5**), is able to remove 34 mg·L^–1^ of As. Teabags submerged in the solution under agitation (**3**) removed more As from water than the teabags submerged in a still solution (**4**). During the MIO-NP embedding process, the Bemliese teabags are immersed in a NaOH solution, which facilitates nanoparticle attachment but can partially compromise the integrity of the cellulose fibers under agitation. This loss of integrity occurs during the embedding step and not during subsequent As-removal experiments conducted under still conditions. As such, the teabags embedded under agitation partially lost their structural integrity, meaning the cellulose in the teabags began to break down or disintegrate, thus rendering them useless for the purposes of creating a “teabag” that can carry eggshells and remain stable under agitation, and woven teabags may be more resistant to breakdown. MIO-NP embedded teabags immersed for 1 h without agitation (4) removed the most As, reducing the concentration from 35 to 6.8 mg·L^–1^, which is an 81% reduction in As content. An increase in the amount of time the teabag was left in the NaOH embedding solution led to uneven distribution of MIO-NP’s and a reduction in the overall effectiveness of the adsorption process. Furthermore, prolonged exposure to the MIO-NP solution can cause the particles to aggregate,^36^ forming larger clusters. These larger aggregates have a lower surface area-to-volume ratio, leading to a relatively lower number of active sites available for As adsorption.^35^

To evaluate the As removal efficiency of the teabag, we calculated the adsorption capacity of individual and combined components (Figure 5) in mg of NaAsO_2_ removed per g of adsorbent (mg·g^–1^). A 6 g Bemliese teabag alone removes up to 13 mg·L^–1^ of NaAsO_2_, corresponding to a capacity of 2.2 mg·g^–1^. When combined with 5 g of eggshells, the system removes 17 mg·L^–1^ of NaAsO_2_, yielding a combined capacity of 1.6 mg·g^–1^ based on the total adsorbent mass (11 g). Embedding MIO-NPs into the teabag significantly increases As removal, as the teabag with MIO-NPs removes 28 mg·L^–1^ (4.7 mg·g^–1^). The highest-performing configuration is a teabag embedded with MIO-NPs and filled with eggshells, which removes 34 mg·L^–1^ of NaAsO_2_, with a combined adsorbent mass of 11 g, giving a maximum capacity of 3.1 mg·g^–1^. Teabags immersed in a 35 mg·L^–1^ NaAsO_2_ solution reduced the concentration to 0.69 mg·L^–1^ after 6 h, a >98% reduction, equivalent to 34 mg·L^–1^ of NaAsO_2_ removed. At more typical environmental concentrations (∼3 mg·L^–1^), equilibrium is reached within 360 min, reducing the concentration of As to ∼0.1 mg·L^–1^, making this system practical for both environmental and commercial water purification applications.

**Figure 5:**
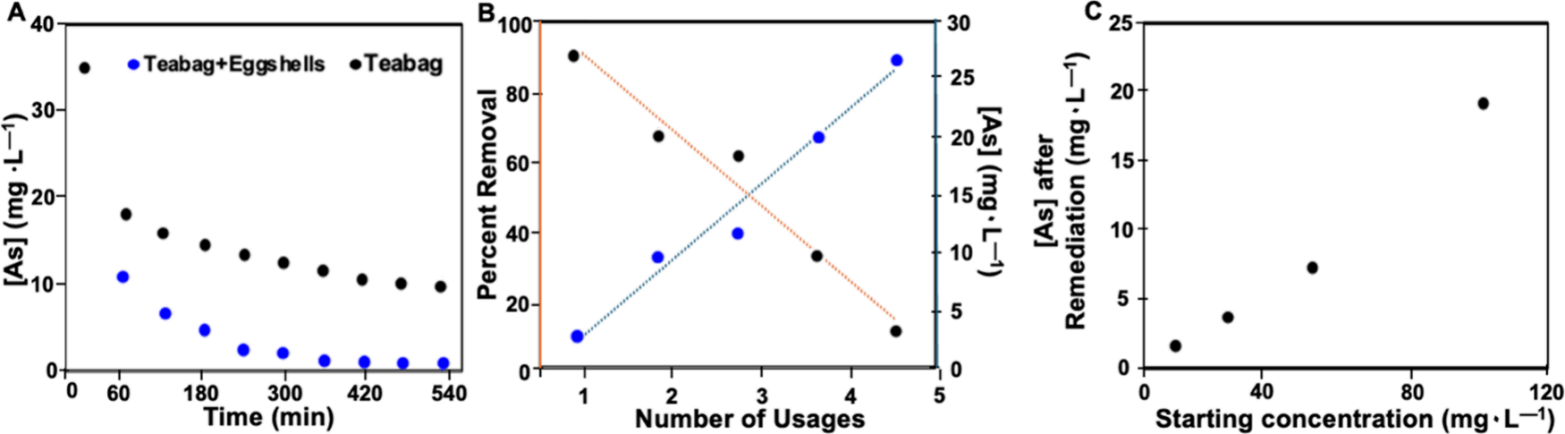
(A) Plot of [As] vs time for a teabag embedded with MIO-NP’s and the embedded teabag filled with eggshells. (B) As removal efficiency after repeated uses of the MIO-NP embedded teabag filled with eggshells. Trials were performed with 50 mL of 35 mg·L^–1^ of NaAsO_2_ solution. (C) Resulting [As] in solution after treatment with the MIO-NP embedded teabag and eggshells at varying starting [As] in 1 L of solution.

The teabags were tested for the number of times each individual teabag could be resubmerged into NaAsO_2_ spiked solution and continue to effectively remove As (Figure 5). After each use, the teabag was gently rinsed with DI H_2_O to remove loosely bound NaAsO_2_, then rinsed in a 0.1 M NH_4_OH solution to desorb adsorbed As species from the surface of the MIO-NPs. This alkaline washing step helps to regenerate the active sites of the MIO-NPs by disrupting the As–Fe binding interactions, restoring partial adsorption capacity. The teabag was subsequently dried in an oven at 150° before being reused in a 50 mL solution containing 35 mg·L^–1^ of NaAsO_2_. Teabags were able to be reused 5 times, after which the continuous drying in the oven began to char the eggshells inside the teabag, and the eggshells began to release a yellow color into the spiked solution, interfering with the spectrometer readings. For each time the teabag was reused, the removal efficacy decreased by ∼19%. Reuse experiments were performed to demonstrate the theoretical reusability of the teabag system and are not intended to represent a requirement for field deployment. Given the low material cost of each teabag, a single-use operation is sufficient to maintain cost-effectiveness for practical groundwater remediation applications. Still, after being used once, a used teabag would be able to remove ∼80% of NaAsO_2_ from 50 mL of a 35 mg·L^–1^ spiked solution. For instance, starting from an original concentration of 3 mg·L^–1^, three uses of the same teabag would still decrease the concentration to ∼5 μg·L^–1^, below the WHO toxicity threshold of 10 μg·L^–1^.

The estimated per-unit cost of the remediation teabag is low compared with traditional remediation methods. A single 6 g Bemliese teabag costs approximately 0.04 USD, while the Fe salts and PVP required to synthesize and embed the MIO-NPs contribute approximately 0.03 USD per teabag. Eggshells are locally sourced waste materials and therefore contribute negligible marginal cost. At environmentally relevant concentrations, a single teabag can treat approximately 1 L of As-contaminated water while still achieving ∼81% overall removal efficiency and can be reused up to five times to further decrease As concentrations. This corresponds to an effective treatment cost of approximately 0.07 USD per liter, supporting the practicality of this approach for decentralized groundwater remediation in resource-limited settings. Thus, the teabag costs about 300 USD a year to treat drinking water for a family of four, which is cheaper than just the maintenance costs of reverse osmosis, which can reach up to 500 USD.^43^

## Conclusions

An assay that can quantitatively measure As in drinking water at ecologically relevant concentrations from 0.64 – 1300 mg·L^–1^ has been developed. The teabag-based remediation strategy developed in this work is intended for decontaminating groundwater and drinking water systems under near-neutral pH rather than in industrial or high-saline wastewater solutions. The LOD and LOQ were 0.21 mg·L^–1^ and 0.64 mg·L^–1^, respectively. The ATIA assay relies on the formation of AsI_3_, which displays a highly visible yellow color. In addition, a biodegradable teabag removes over 98% of 35 mg·L^–1^ NaAsO_2_ from water in a 50 mL solution or 94% of NaAsO_2_ from a 100 mL solution, and the NaAsO_2_ absorption capacity of these teabags was 34.3 mg·L^–1^. This teabag consists of MIO-NPs embedded in Bemliese, a low-lint cellulose fiber with 5 g·L^–1^ of mechanically ground uncharred eggshells in the bag. Uncharred eggshells can remove ∼96% of NaAsO_2_ in a 50 mL spiked solution, decreasing the concentration from 35 mg·L^–1^ to 1.4 mg·L^–1^. The MIO-NP modified teabags alone can remove 34.3 mg·L^–1^ As from water. Teabags left in solution for 6 h were found to be most effective, and they decreased the concentration from 35 to 0.69 mg·L^–1^, corresponding to a removal of 34.31 mg·L^–1^. At a high NaAsO_2_ concentration of 3 mg·L^–1^, three uses of the same teabag in a solution would decrease the concentration to 8 μg·L^–1^, demonstrating the reusability of these bags. At the 227 μg·L^–1^, a number representing the [NaAsO_2_] of wells in Bangladesh,^37^ only one teabag is needed to reduce the concentration of 50 mL of water to the acceptable concentration of 4.54 μg·L^–1^, below the WHO toxicity standard of 10 μg·L^–1^. Taken together, the combination of new methods for detection and removal provides scalable, reusable, and cost-effective solutions to the global health challenges posed by As contamination in drinking water. Future work will explore alternative cotton fibers, commercial teabags, or other biodegradable substrates to further improve the adsorption efficiency and mechanical stability. In particular, materials that better tolerate alkaline embedding conditions and mechanical agitation will be investigated to mitigate the integrity loss observed during NaOH-assisted MIO-NP embedding. Optimizing the fiber structure or surface chemistry of these materials may increase the As removal capacity while maintaining reusability. While reuse is not required for cost-effective deployment, future studies may also evaluate simplified regeneration approaches or single-use designs optimized for field conditions, thereby minimizing handling steps.

## Supplementary Material



## Notes

Additional experimental details; full ATIA calibration data; interference studies with Cr­(III), Ni­(II), and Mn­(II); full SEM/EDX images and elemental maps of used teabags and eggshells; thermodynamic analysis and Van’t Hoff plots; cost analysis; and supplementary tables and figures. (PDF)
