# Supplementary material for: Detection of Arsenic at Micromolar Concentrations and Remediation of Arsenic from Drinking Water with a Bemliese Teabag
Source: ACS Omega. 2026 Mar 19;11(12):19381–90. doi: 10.1021/acsomega.5c12885 (PMC13044616; doi:10.1021/acsomega.5c12885)
Supplement: Supplementary file 1 [file ao5c12885_si_001.pdf]

Supporting Information

Detection of Arsenic at Micromolar Concentrations and Remediation of Arsenic from Drinking Water with a Bemliese Teabag

Vick J. Tan[a,b,c*], Manuel A. Lema[a,d,e], Keidy L. Matos[a,d,e], Adam B. Braunschweig[a,d,e*]

a) Advanced Science Research Center, Graduate Center, City University of New York, 85 St. Nicholas Terrace, New York, NY 10031, USA.

b) EP Academy, 12310 Singletree Lane, Eden Prairie, Minnesota, MN 55344, USA.

c) Scarsdale High School, 1057 Post Road, Scarsdale, NY 10583, USA.

d) Department of Chemistry, Hunter College, 695 Park Avenue, New York, NY 10065, USA.

e) PhD Program in Chemistry, Graduate Center, City University of New York, 365 5th Avenue, New York, NY 10016, USA.

Email: abraunschweig@gc.cuny.edu, vicktanjl@gmail.com

Keywords: Arsenic, Arsenic Detection, Bioremediation, Sustainable, Water Treatment

Table of Contents

1. *Optimized Leucomalachite Green Method Detection of As* 3
   A: General Methods: 3
   B: UV-VIS Spectra of the Optimized Leucomalachite Green Method: 4
2. *Arsenic Tri-Iodide Assay (ATIA) Development* 4
   A: General Methods: 4
   B: UV-VIS Spectra of the ATIA 5
   C. Regression ANOVA Statistics for the ATIA 8
   D. Cost Analysis for the ATIA 9
3. *Teabag Development* 10
   A. Teabag Enthalpy and Entropy Calculations: 14
4. *Scanning electron microscopy and energy-dispersive X-ray Analysis* 15
   A: General Methods: 16
5. *Materials/Methods* 23
   A. Preparation of Iron Oxide Nanoparticles (MIO-NPs): 25
   B. Preparation of Eggshells: 26
   C. Preparation of Teabags: 27
   D. Apparatus: 27
   E. Reagents: 27
6. *References* 30

1. Optimized Leucomalachite Green Method Detection of As

A: General Methods

All reagents and starting materials were purchased from Sigma-Aldrich or VWR and used without further purification. The instrument used was V-660 JASCO UV-Vis/NIR Spectrophotometer (JASCO, V-600 Series), and absorbance was measured from 700 nm – 300 nm using a quartz cuvette with a path length of 1.0 cm. For the Leucomalachite green method[1], a sodium meta-arsenite ($NaAsO_2$) (As) sample (6 mL) was transferred to a glass vial. Potassium iodate (1% w/v, 1 mL, 0.0467 mmol) and hydrochloric acid (1 M, 0.5 mL, 0.5 mmol) were added, and the mixture was gently shaken and left for 2 min. Leucomalachite Green dye was added (0.05% w/v, 0.5 mL, 0.000755 mmol), followed by sodium triacetate buffer (13.6% w/v, 2 mL, 1.30 mmol). The mixture was gently shaken and left for 5 min. The absorbance was measured at 443 nm against the blank of water, potassium iodate (1% w/v, 1 mL, 0.0467 mmol), sodium triacetate buffer (13.6% w/v, 2 mL, 1.30 mmol), and hydrochloric acid (1 M, 0.5 mL, 0.5 mmol).

B: UV-VIS Spectra of the Optimized Leucomalachite Green Method:

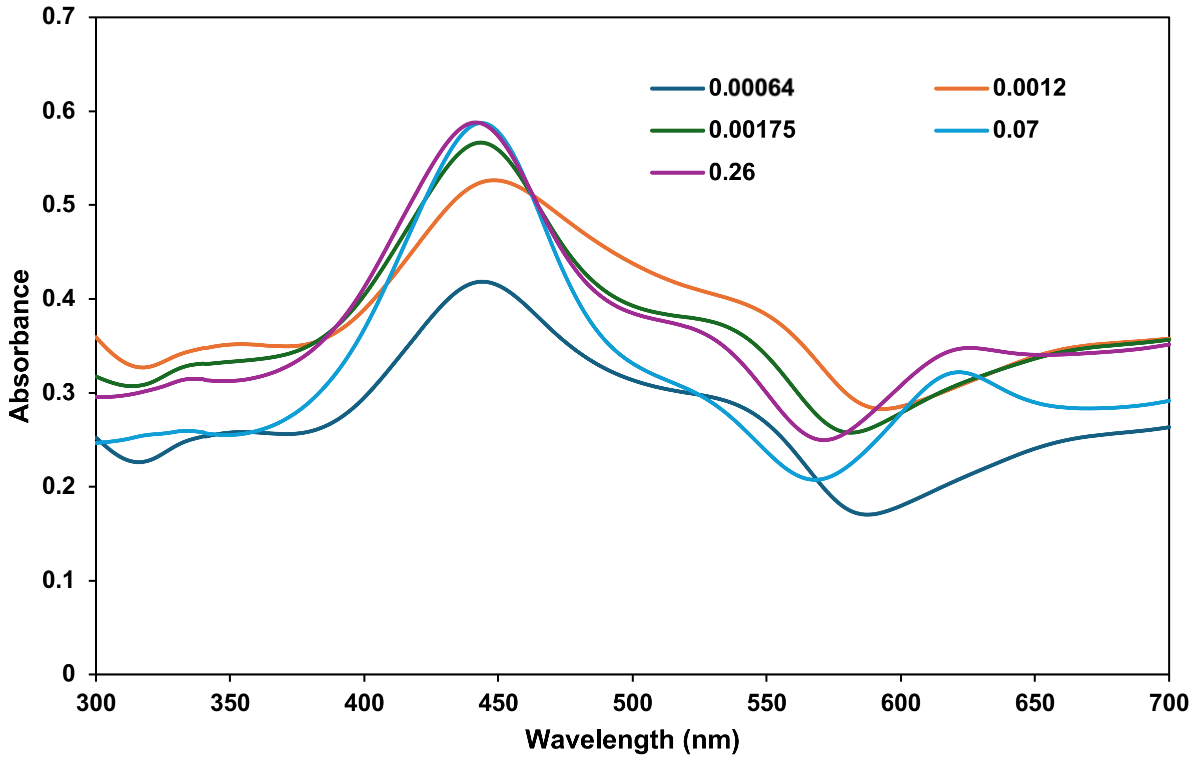

Figure S1: UV-VIS Spectrum of the Leucomalachite Green Assay from 300-700 nm at [$NaAsO_2$] at a concentration of 0.64 μg·L^{-1}, 1.2 μg·L^{-1}, 1.7 μg·L^{-1}, 70 μg·L^{-1}, 260 μg·L^{-1}.

2. Arsenic Tri-Iodide Assay (ATIA) Development

A: General Methods:

The solution for the AITA was prepared by sodium meta-arsenite ($NaAsO_2$, 6 mL), potassium iodate (KIO_3, 1% w/v, 1 mL, 0.0467 mmol), and hydrochloric acid (HCl, 1 M, 0.5 mL, 0.500 mmol). The number of reagents that developed the strongest color was: sodium meta-arsenite ($NaAsO_2$, 6 mL), potassium iodate (KIO_3, 1% w/v, 0.5 mL, 0.0234 mmol), and hydrochloric acid (HCl, 1 M, 0.25 mL, 0.250 mmol), which was gently shaken and left for 2 min.

B: UV-VIS Spectra of the ATIA

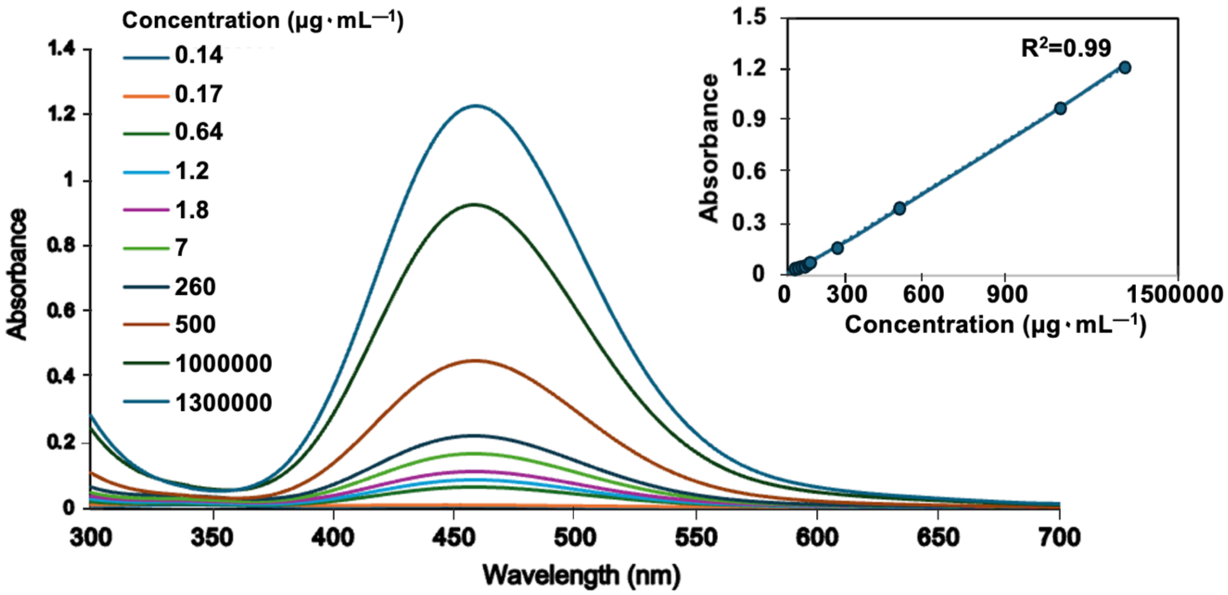

Figure S2: UV-Vis Spectrum of the ATIA from 300-700 nm at [$NaAsO_2$] of 0.14, 0.17, 0.64, 1.2, 1.8, 7, 260, 500, 1000000, and 1300000 µg·mL^{-1}. Inset graph portrays the relationship between [$NaAsO_2$] and absorbance measured.

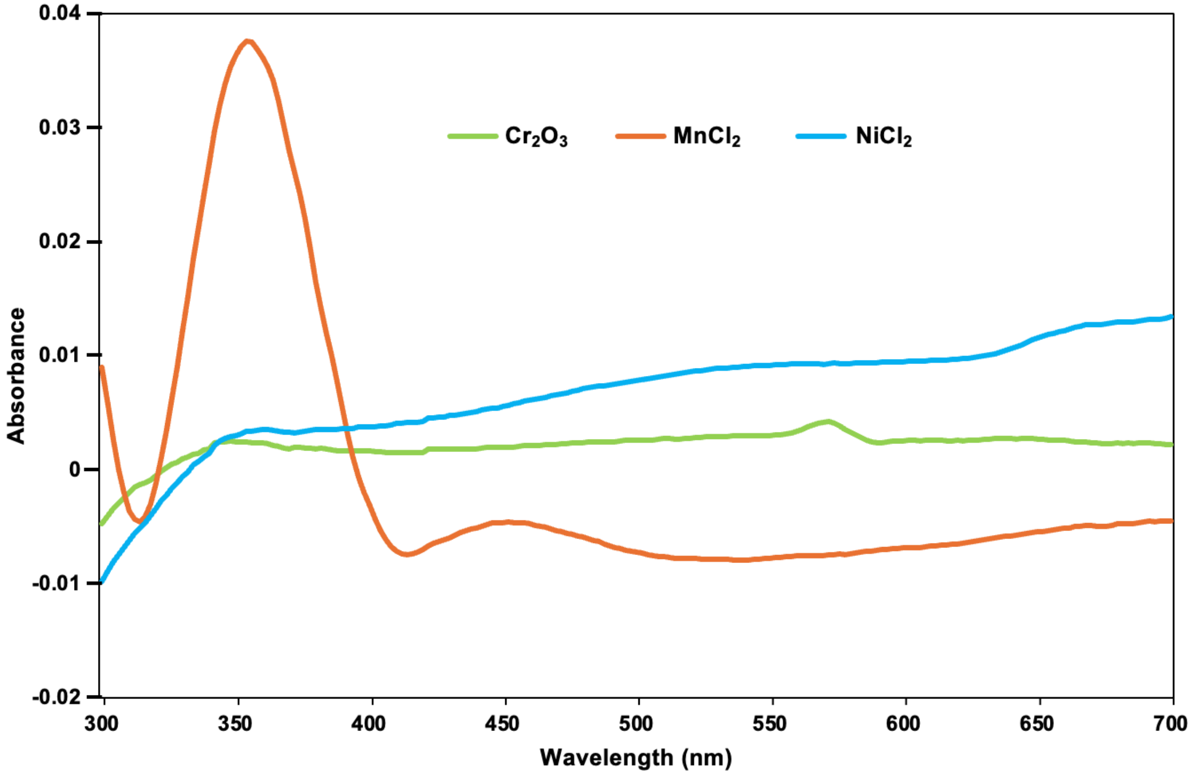

Figure S3: UV-VIS Spectrum after being tested with the ATIA for 3 mL each of chromium(III) oxide (Cr_2O_3, 0.05 mg·L^{-1}, 3 mL, 0.00031 mmol), nickel(II) chloride ($NiCl_2$, 0.5 μg·L^{-1}, 3 mL, 2.34 × 10^{-5} mmol), and manganese(II) chloride ($MnCl_2$, 0.1 mg·L^{-1}, 3 mL, 0.00237 mmol) from 300–700 nm.

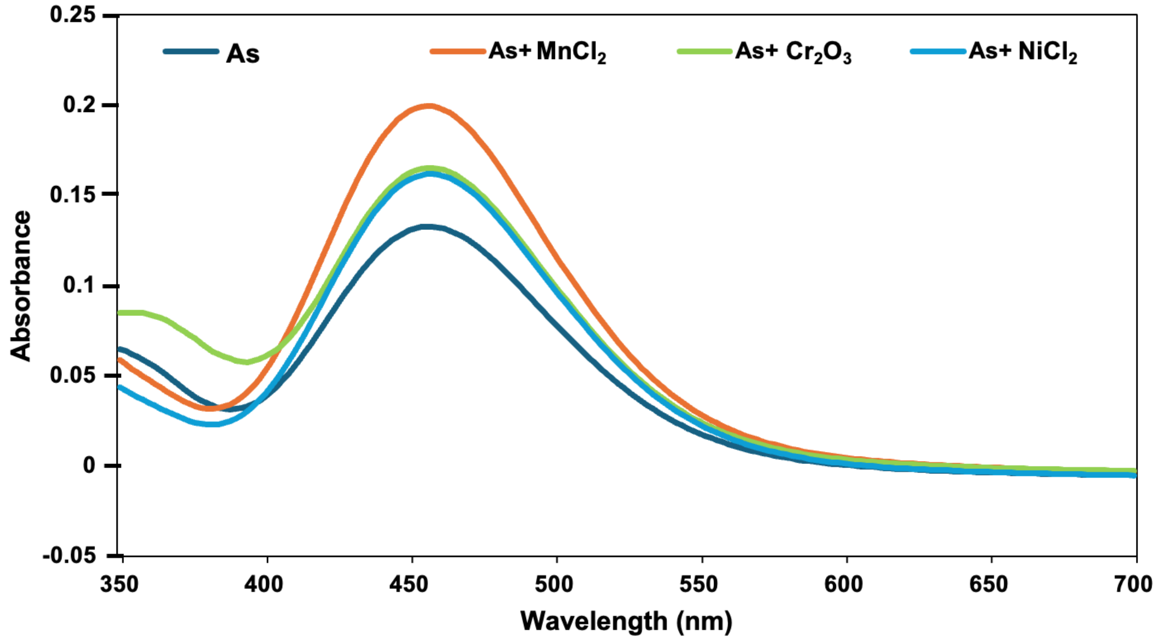

Figure S4: UV-VIS Spectrum of a $NaAsO_2$ solution at 35 mg·L^{-1} (0.378 mmol) spiked with 3 mL each of (Cr_2O_3, 0.05 mg·L^{-1}, 3 mL, 0.00031 mmol), ($NiCl_2$, 0.5 μg·L^{-1}, 3 mL, $2.34 \cdot 10^{-5}$ mmol), and ($MnCl_2$, 0.1 mg·L^{-1}, 3 mL, 0.00237 mmol) after being tested with the ATIA from 300–700 nm.

C. Regression ANOVA Statistics for the ATIA

A regression analysis of As concentration at multiple different concentrations was performed based on absorbance values obtained from the ATIA, in order to determine the significance of the linear relationship between As concentration and absorbance in the ATIA assay. The data collected from concentrations of 0.14 $\mu g{\cdot}L^{-1}$ to 1.3 $mg{\cdot}mL^{-1}$ were fitted to a nonlinear regression model to describe the concentration–absorbance relationship. ANOVA results confirmed a statistically significant correlation between absorbance and concentration when quantified using the ATIA ($p < 0.05$). The regression curve demonstrated a plateau at higher concentrations, consistent with adsorption saturation. A R^2 value of > 0.99 supported the model's goodness of fit, validating the assay's ability to quantify As.

ANOVA

	df	*SS*	*MS*	*F*	*Significance F*
Regression	1	4.03464E-05	4.0346E-05	245688026	4.0702E-09
Residual	2	3.28436E-13	1.6422E-13		
Total	3	4.03464E-05			

	Coefficients	*Standard Error*	*t Stat*	*P-value*	*Lower 95%*	*Upper 95%*	*Lower 95.0%*	*Upper 95.0%*
Intercept	4.4965E-07	3.48107E-07	1.29171442	0.32559513	-1.04813E-06	1.9474E-06	-1.04813E-06	1.9474E-06
concentration	9.3903305	0.000599086	15674.4386	4.0702E-09	9.387752846	9.39290816	9.387752846	9.39290816

Figure S5: Regression ANOVA analysis of the ATIA.

D. Cost Analysis for the ATIA

The cost per assay was determined by adding by the cost of the reaction chamber and the reagents -- hydrochloric acid and potassium iodide.

- Vial: $0.23
- 0.5 mL (1% w/v) Potassium Iodide: $0.0029
- 0.25 mL 1M HCL: $0.007

Total cost per test: $0.24

When comparing the cost per test for the ATIA developed with industrial test kits, our average cost per test ($0.24) is ~ 21x cheaper than the average cost per test for industrial testing kits ($5.06). Furthermore, since the vial can be reused, the potential cost per test could drop to <$0.01 per test.

3. Teabag Development

Eggshells were prepared following the methods outlined in "Section 7. Materials and Methods". Then, 50 mL of spiked solution was treated with the ground eggshells. The solution was then left to rest for an hour and decanted and filtered to remove the ground eggshells, after which it was measured by UV-VIS at the peak wavelength of 458 nm.

Table S1: Absorbance of $NaAsO_2$ solution treated with eggshells at 458 nm.

Eggshell mass (g)	Absorbance of charred eggshells (200 °C)	Absorbance of uncharred eggshells (25 °C)
0.5	— 0.106	0.0131
1.0	— 0.140	0.0158

Note that absorbance is negative for the charred eggshells as a result of charring process darkening the eggshells in the control solution and leading to the release of a yellow color when the eggshells were placed into the spiked solution.

10 teabags (~ 6 g) without the addition of eggshells were added into ($NaAsO_2$, 35 mg·L^{-1}, 50 mL, 11.9 mmol) in different beakers. Then, an aliquot was taken from each beaker at 0, 60, 120, 180, 240, 300, 360, 420, 480, and 540 minutes. After each time was reached, an aliquot of 3 mL was taken then added to a vial where the ATIA was performed and concentration was determined.

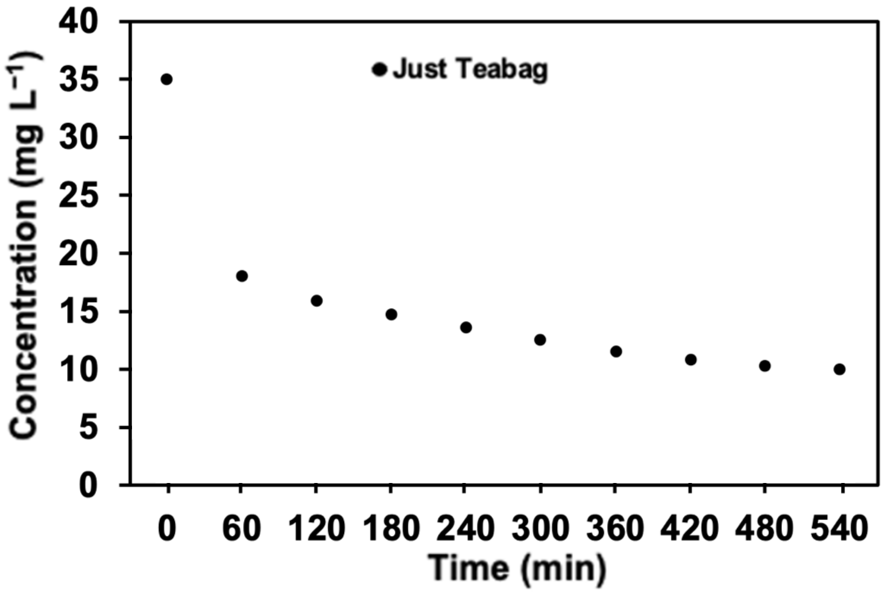

Figure S6. [$NaAsO_2$] in solution, as determined by the ATIA, after treatment with a MIO-NP embedded teabag (~ 6 g) submerged into ($NaAsO_2$, 35 mg·L^{-1}, 50 mL, 11.9 mmol) from 0 - 540 minutes.

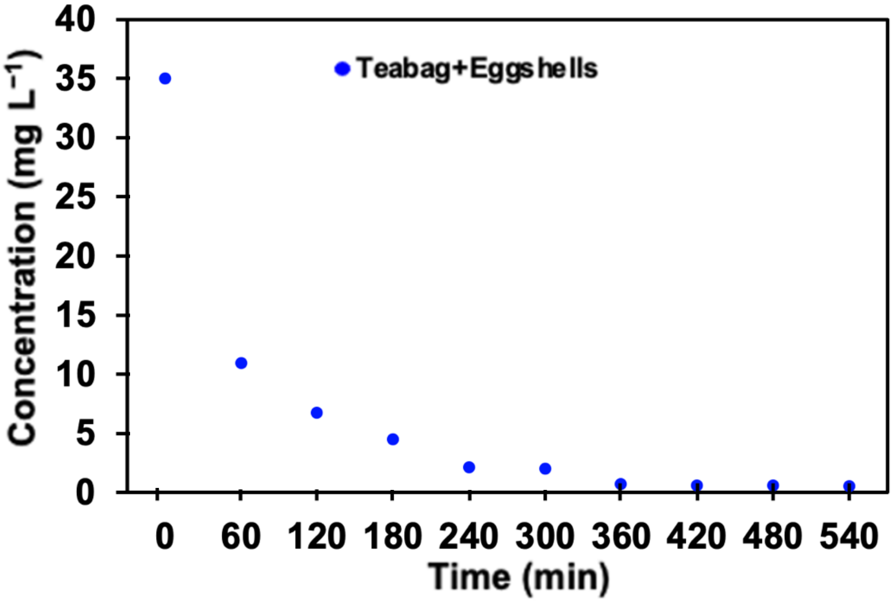

Figure S7. [$NaAsO_2$] in spiked solution, as determined by the ATIA, after treatment with a MIO-NP embedded teabag and uncharred eggshells (~ 11 g) submerged into ($NaAsO_2$, 35 mg·L^{-1}, 50 mL, 11.9 mmol) from 0 - 540 minutes.

Teabags were tested for reusability. After each use, the teabag was gently rinsed with 200 mL DI H_2O to remove loosely bound $NaAsO_2$, then rinsed in ammonium hydroxide (NH_4OH, 0.1 M, 200 mL, 20 mmol) to desorb the strongly adsorbed As species from the surface of the MIO-NPs. This alkaline washing step helps regenerate the active sites of the MIO-NPs by disrupting the As–Fe binding interactions, restoring partial adsorption capacity. Then, the teabag was dried in an oven at 150 °C before being reused in a solution ($NaAsO_2$, 35 mg·L^{-1}, 50 mL, 11.9 mmol). Teabags were able to be reused 5 times, after which the continuous drying in the over began to char the eggshells inside the teabag, and the eggshells began to release a yellow color into the spiked solution, interfering with the spectrometer readings.

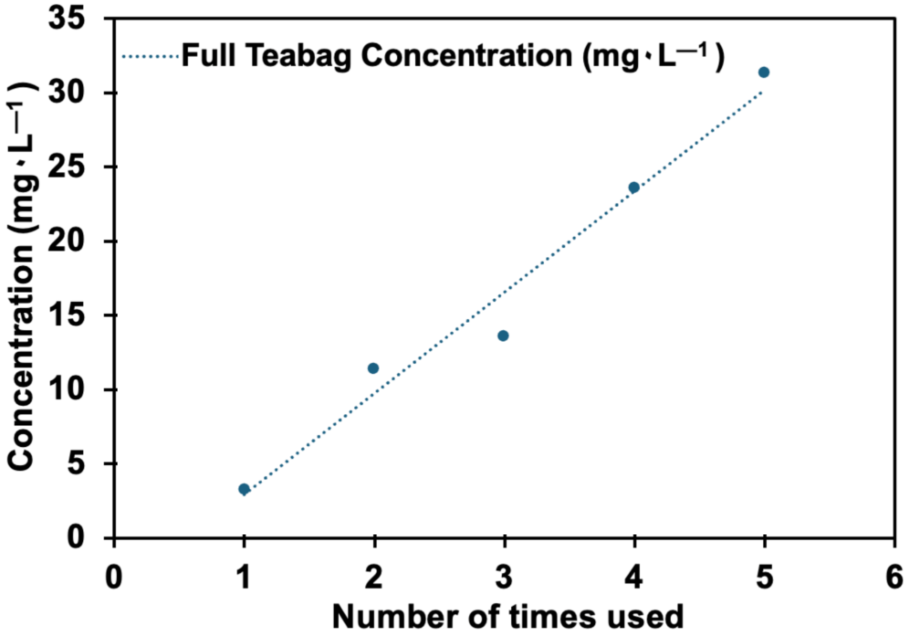

Figure S8. [$NaAsO_2$] in spiked solution, as determined by the ATIA, after treatment with a MIO-NP embedded teabag and uncharred eggshells (~ 11 g). One teabag was used and submerged in ($NaAsO_2$, 35 mg·L^{-1}, 50 mL, 11.9 mmol) solution 5 times each for 6 hours.

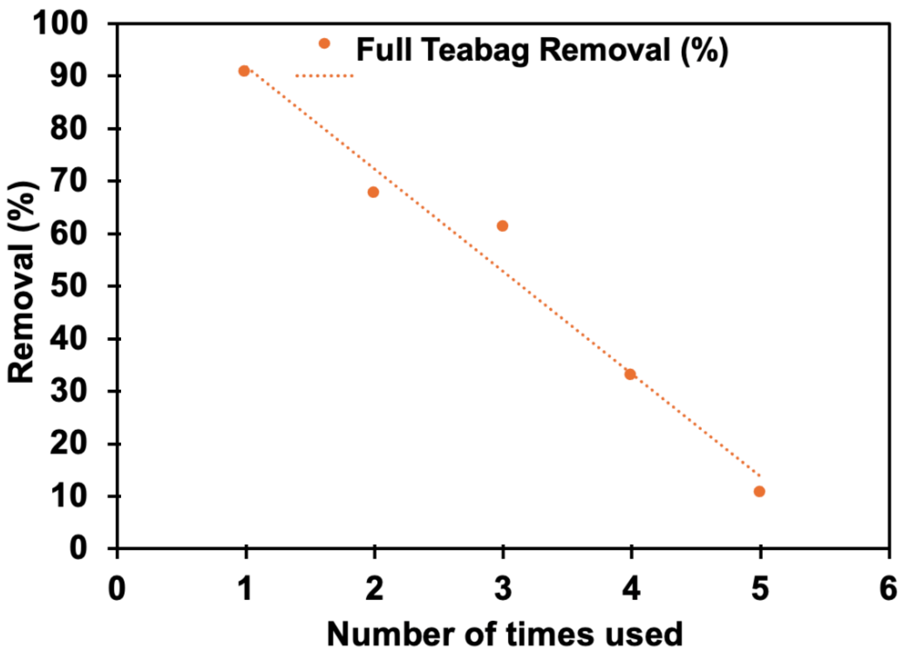

Figure S9. Removal of $NaAsO_2$ from a spiked solution, as determined by the ATIA, after treatment with a MIO-NP embedded teabag and uncharred eggshells (~ 11 g). One teabag was used and submerged in ($NaAsO_2$, 35 $mg \cdot L^{-1}$, 50 mL, 11.9 mmol) solution 5 times each for 6 hours.

The ability of teabags to remove $NaAsO_2$ were also tested at higher concentrations of As (up to 100 $mg \cdot L^{-1}$), which are commonly found near anthropogenic sources such as mines[2].

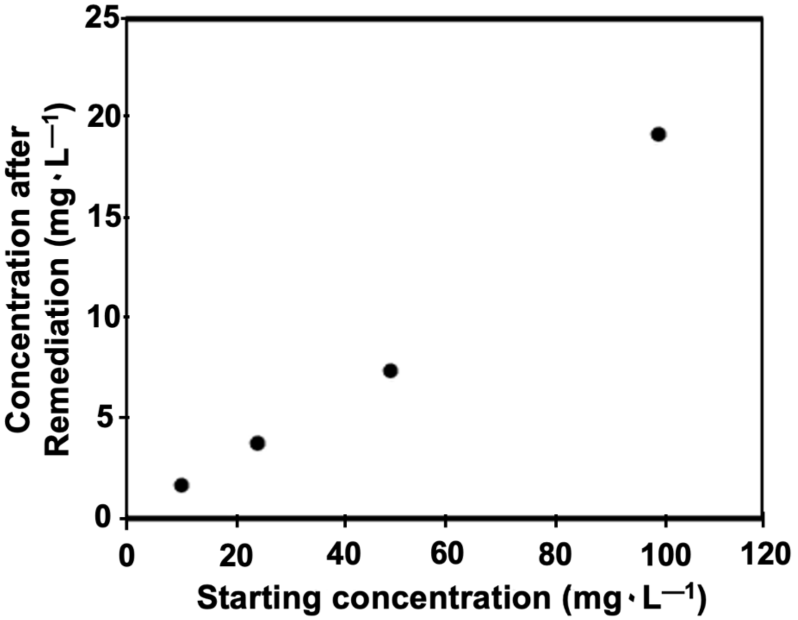

.

Figure S10. Concentration of $NaAsO_2$ from a spiked solution after treatment with a MIO-NP embedded teabag and uncharred eggshells (~ 11 g). One teabag was used and submerged in ($NaAsO_2$, 35 mg·L^{-1}, 50 mL, 11.9 mmol) solution 5 times each for 6 hours.

A. Teabag Enthalpy and Entropy Calculations:

The amount of As adsorbed per unit mass of adsorbent at equilibrium, denoted as q_e, was calculated using the equation:

$$q_e = \frac{(C_i \ - \ C_e) \ * \ \mathrm{V}}{M}$$

Where C_i is the initial As concentration (mg·L^{-1}), C_e is the equilibrium concentration (mg·L^{-1}), V is the solution volume (L), and M is the mass of the adsorbent (g). A plot of the natural logarithm of q_e against the natural logarithm of was C_e constructed, and the intercept of this line represents ln K_f, where K_f is the equilibrium constant. At room temperature (303.15 K), the equilibrium constant was determined to be 0.043, while at 323.15 K it increased to 0.20.

Thermodynamic parameters were then obtained using the Van't Hoff equation[3]:

$$\ln\left(\frac{K_f(T_2)}{K_f(T_1)}\right) = -\frac{\Delta \mathrm{H}}{R}\left(\frac{1}{T_2} - \frac{1}{T_1}\right)$$

where R is the universal gas constant (8.31 J·mol^{-1}·K^{-1}), and T_1 and T_2 are the absolute temperatures (K). Substituting the experimental values yielded an enthalpy change (ΔH°) of 20 kJ·mol^{-1}**,** indicating that the adsorption process is endothermic.

The Gibbs free energy change, ΔG°, was then calculated using the relation:

$$\Delta G^{\circ} = -\mathrm{RTln}(K_f)$$

At 303.15 K, ΔG° was 8.57 kJ·mol^{-1}, while at 323.15 K, ΔG° decreased to 6.54 kJ·mol^{-1}, reflecting a more favorable adsorption process at higher temperature. Finally, the entropy change, ΔS^{o}, was obtained from the expression:

$$\Delta \mathrm{S} = \frac{\Delta \mathrm{H} - \Delta G_2}{T_2}$$

Where $\Delta G^{o}{}_2$ is the Gibbs free energy at 323.2 K. The calculated ΔS° was 41.4 J·mol^{-1}·K^{-1}**.** Together, the positive ΔH° and ΔS° values, suggest that the adsorption mechanism is chemisorption.

Table S2: Thermodynamic parameters of Arsenic adsorption at different temperatures.

Temperature (K)	K_f	ΔG° (kJ·mol^{-1})	ΔH° (kJ·mol^{-1})	ΔS° (J·mol^{-1}·K^{-1})
**303.15**	**0.043**	**8.57**	**20**	**41.4**
**323.15**	**0.20**	**6.54**	**20**	**41.4**

4. Scanning electron microscopy and energy-dispersive X-ray Analysis

A: General Methods:

Eggshell fragments (approximately 4 x 4 mm) and prepared teabag samples were cut down to the same size and collected for imaging and elemental analysis. Samples were mounted on conductive aluminum stubs (Product No. 16120, Ted Pella, Inc.) and sputter-coated with 5 nm layer of gold. Imaging was performed on a FEI Helios Nanolab 660 focused ion beam–scanning electron microscope (FIB-SEM). Elemental composition and mapping were carried out using an Oxford Instruments X-Max energy-dispersive X-ray spectroscopy (EDS) detector integrated with the SEM.

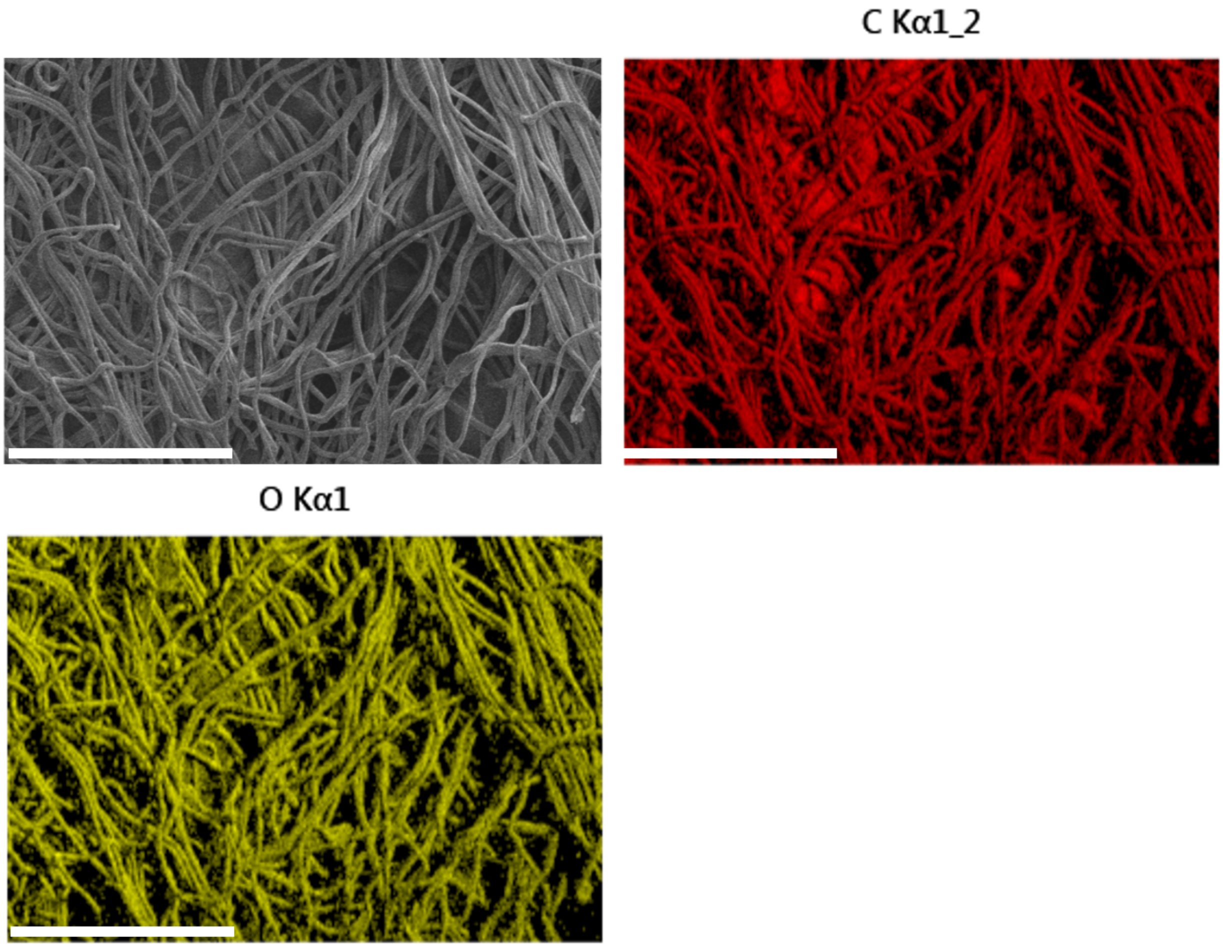

Figure S11. SEM analysis of Bemliese Teabag. Scale bar represents 500 μm.

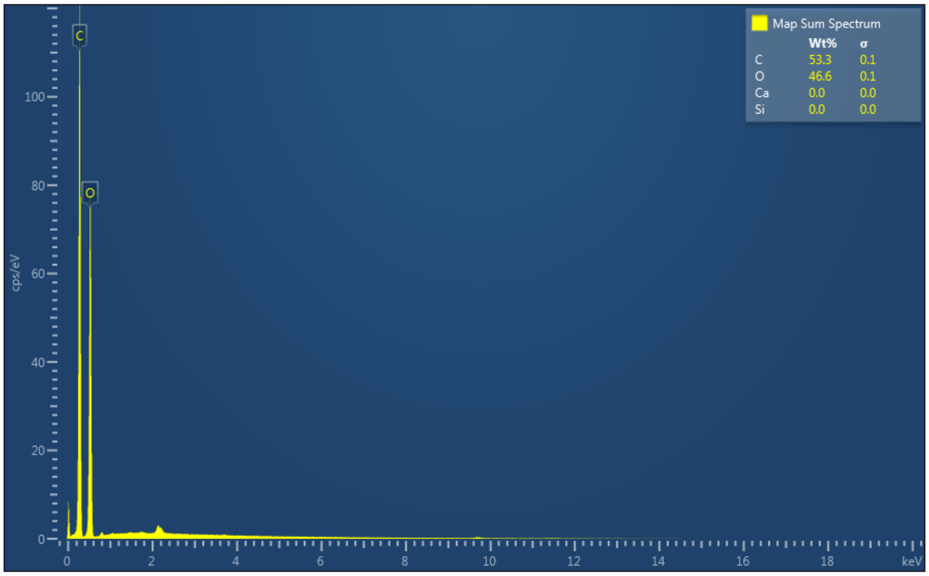

Figure S12. EDX analysis of Bemliese Teabag.

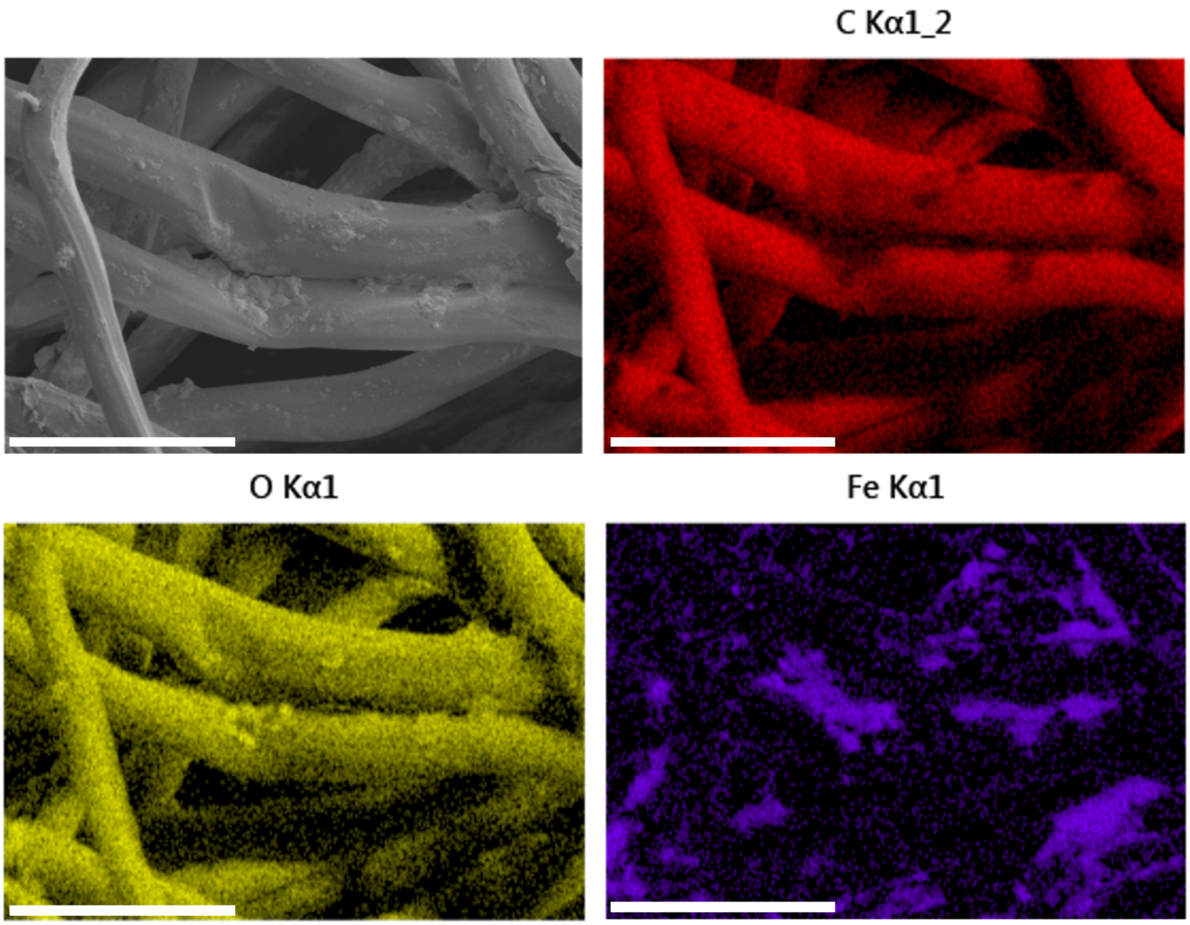

Figure S13. Teabag embedded with MIO-NP's at 1 h without agitation. Scale bar represents 50 μm.

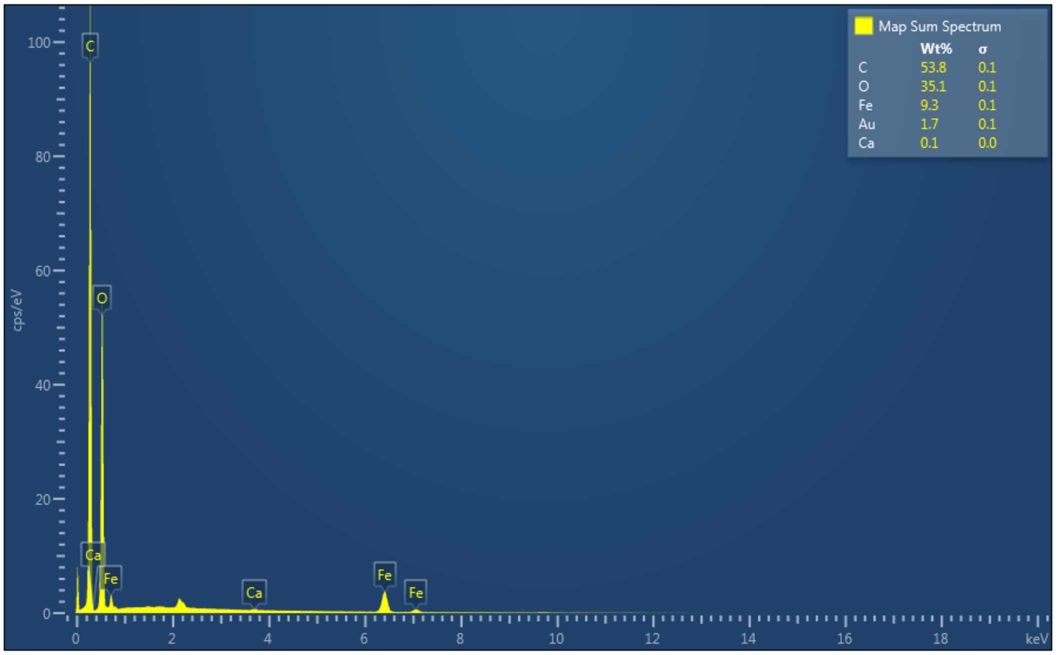

Figure S14. EDX of Teabag embedded with MIO-NP's at 1 h without agitation. Scale bar represents 50 μm.

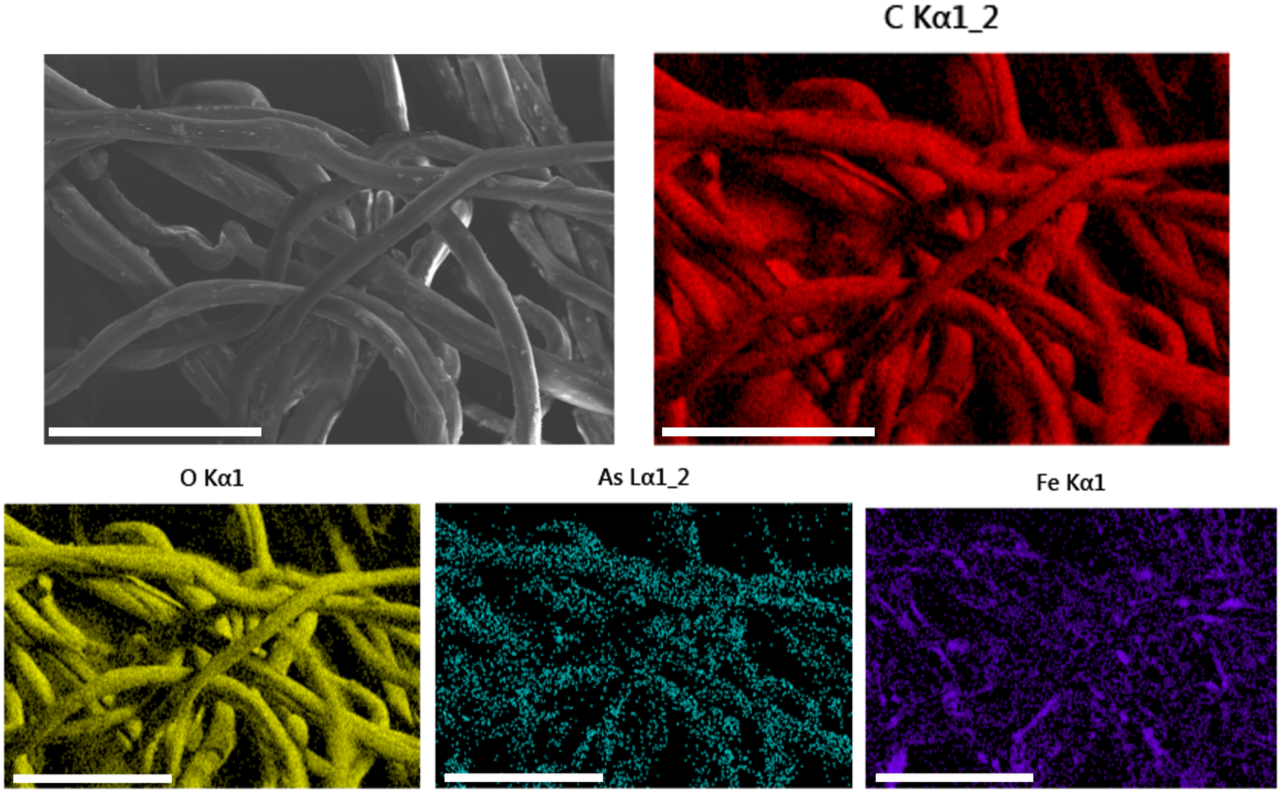

Figure S15. Teabag embedded with MIO-NP's at 1 h without agitation after usage to remove As. Scale bar represents 100 μm.

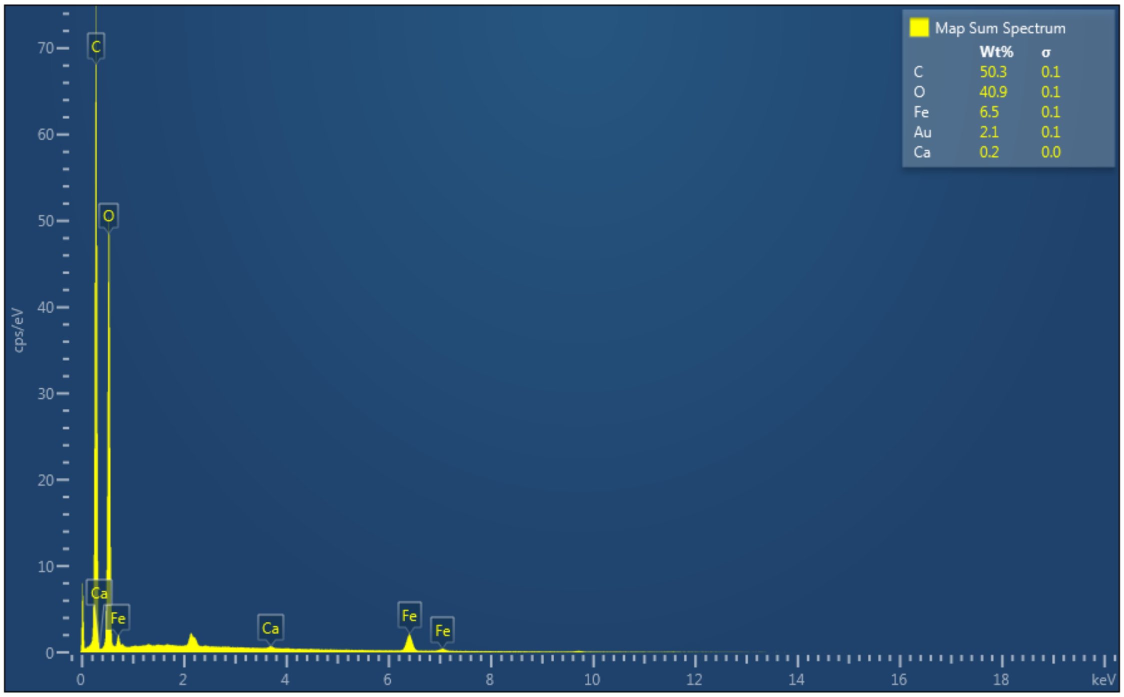

Figure S16. EDX of Teabag embedded with MIO-NP's at 1 h without agitation after usage to remove As.

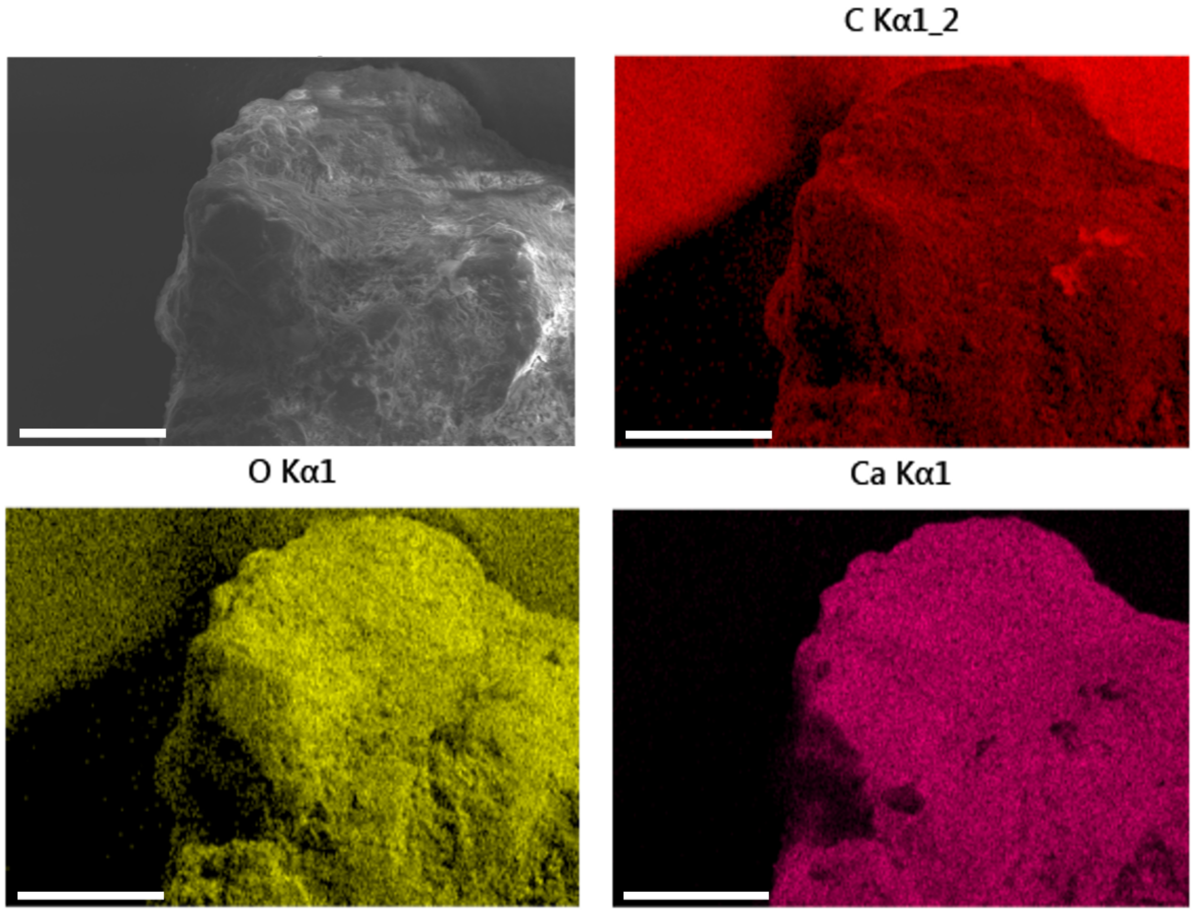

Figure S17. SEM images of Eggshell. Scale bar represents 100 μm.

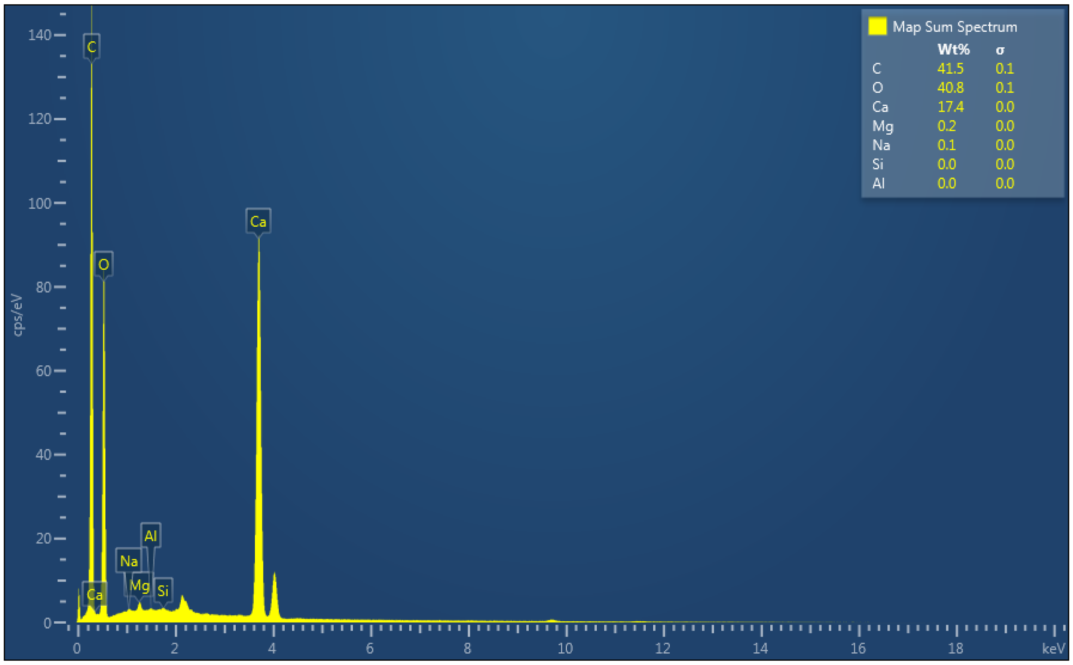

Figure S18. EDX of Unused Eggshell.

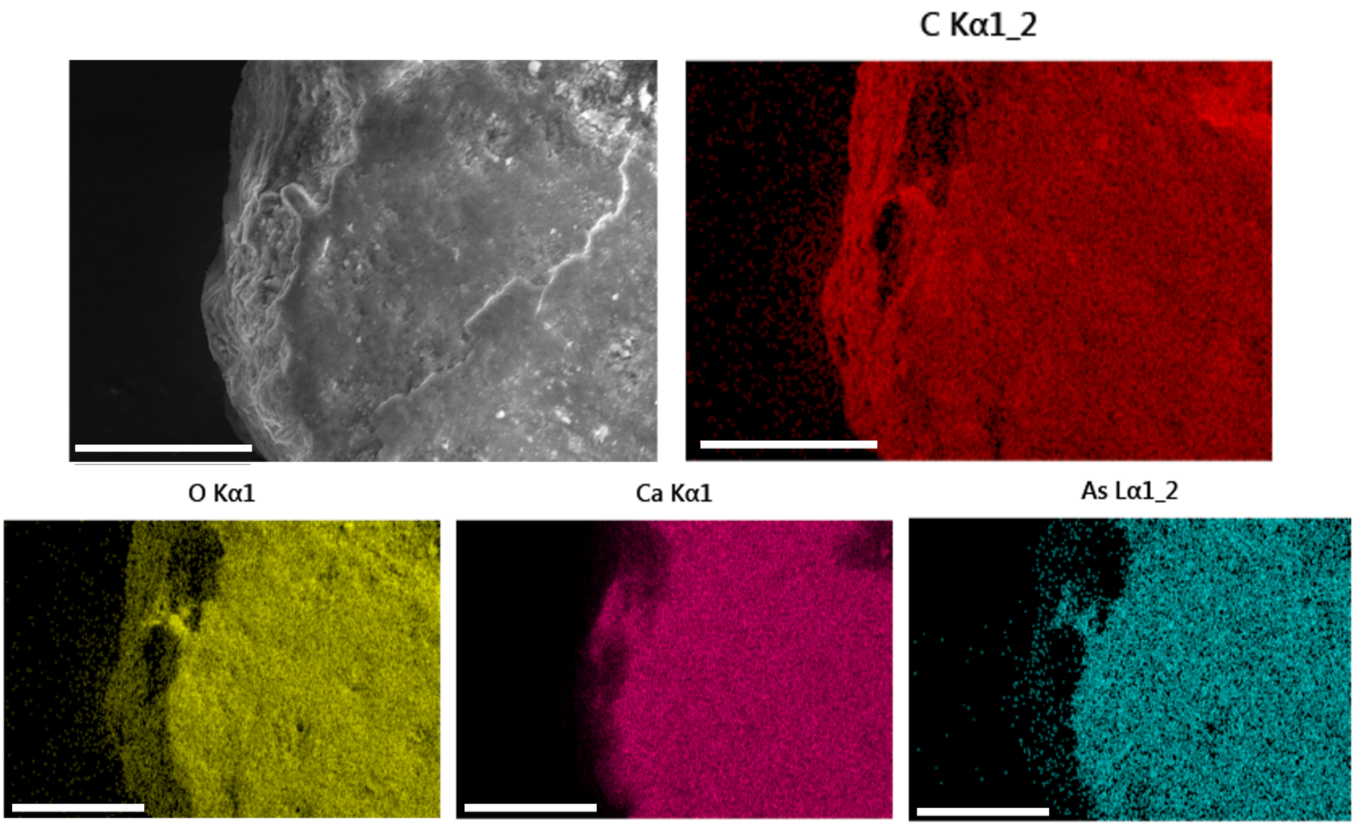

Figure S19. SEM images of Eggshell after being used to remove As. Scale bar represents 50 μm.

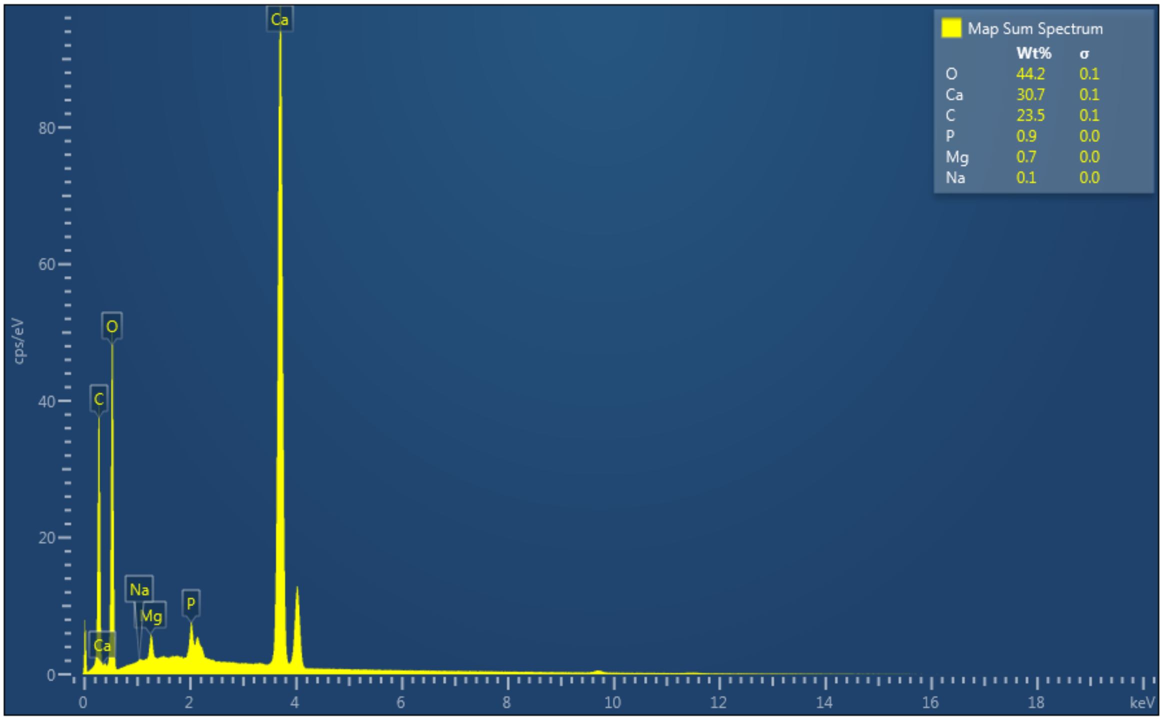

Figure S20. EDX of Eggshell used to remove As.

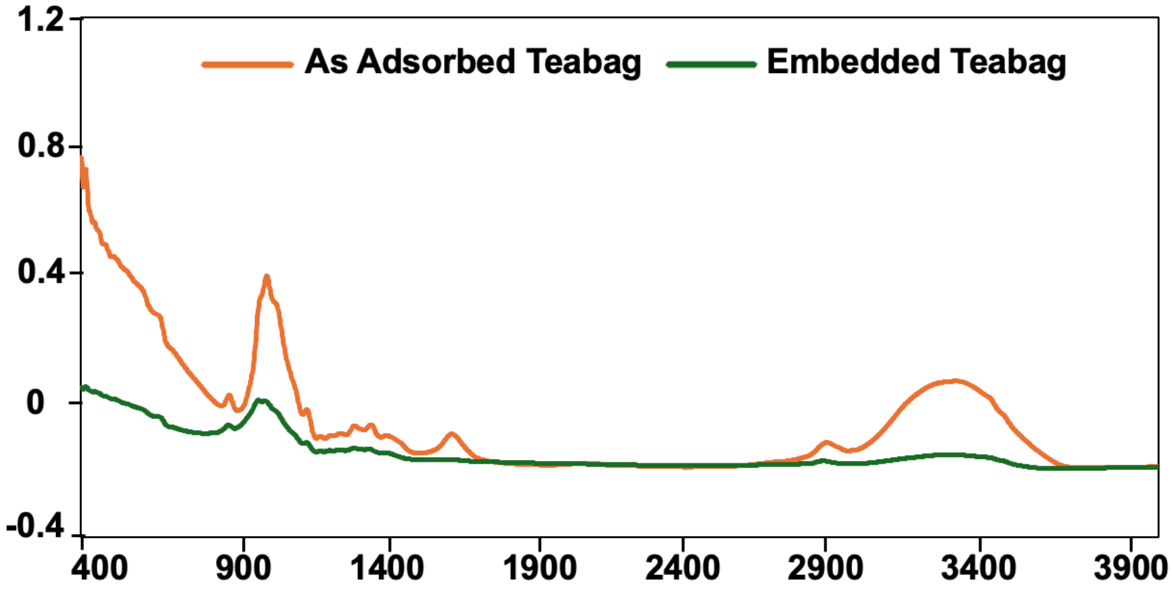

Figure S21. ATR-FTIR spectra (400–4000 cm^{-1}) of the MIO-NP–embedded Bemliese teabag before arsenic exposure (Embedded Teabag) and after arsenic adsorption (As-Adsorbed Teabag). Changes in the O–H stretching region (~3300–3500 cm^{-1}) and the appearance of

features in the ~900–1000 cm⁻¹ region after adsorption are consistent with surface complexation of As(III) on iron-oxide hydroxyl sites.

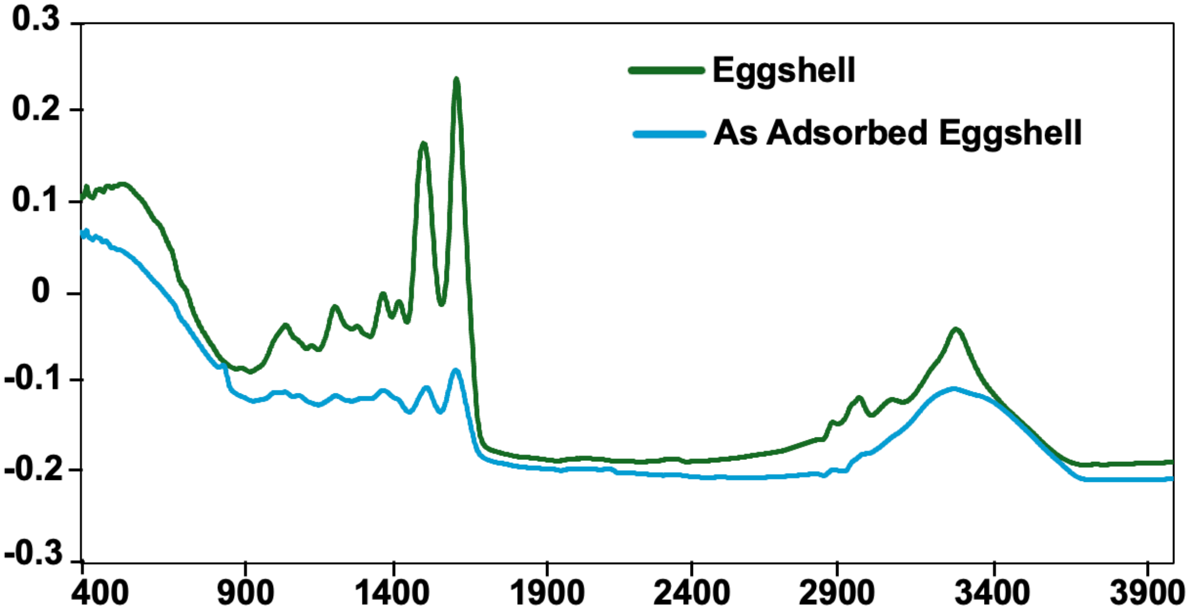

Figure S22. ATR-FTIR spectra (400–4000 cm^{-1}) of uncharred eggshells before and after arsenic adsorption**.** Changes in the carbonate stretching bands (~1400-1500 cm^{-1} and ~870 cm^{-1}) following As exposure indicate interaction between arsenic species and $CaCO_3$-rich binding sites on the eggshell surface.

5. **Materials/Methods**

A. **Preparation of Iron Oxide Nanoparticles (MIO-NPs):**

i. Iron Oxide Nanoparticles were synthesized by adding 5.0 g Iron (II) chloride and 13.5 g Iron (III) chloride in 500 mL of DI H_2O at 60°C under inert Ar atmosphere in a 1L round bottom flask. After 30 minutes of mixing using a stir bar at 900 rpm, 280 mL of Ammonium Hydroxide solution at 28% w/v was injected. Then, 50 mL of 5 M polyvinylpyrrolidone (PVP) was added. After 2 hours of mixing at 900 rpm, the color of the solution turned black, and the black precipitate was filtered out and washed with DI H_2O. Once the MIO-NPs were collected, they were dried overnight by leaving the MIO-NPs on filter paper with constant airflow.

ii. Iron Oxide Nanoparticles were embedded into the Bemliese fabric by adding 25 g of MIO-NPs to 450 mL of DI H_2O and 50 mL of Ammonium Hydroxide solution at 28% w/v in a 1L round bottom flask. Then, 10 teabags were added to the flask, and fully submerged under the solution. After waiting a 1 hour, teabags were removed from the flask and washed in DI H_2O to remove excess unbinded MIO-NPs.

B. Preparation of Eggshells:

i. Preparation of uncharred eggshells: Eggshells were locally sourced. The egg was fully washed in DI H_2O and cleaned to ensure that only the shell and inner membrane remained. The eggshells were left to dry for 24 hours in a fume hood. After which, they were physically broken into smaller pieces by hand. Then, the 5g of eggshells were transferred into the ball mill with one large ball of diameter 100 nm. The ball mill was run for 5 minutes at a 30 Hertz. The eggshells were collected into a beaker and rinsed with H_2O until the H_2O ran clear to remove any small particles and ensure a relatively even particle size. Then, the eggshells were left to dry in a beaker overnight.

ii. Preparation of charred eggshells: Same as preparation of uncharred eggshells, except after physically breaking the eggshell into smaller pieces, they were added into a 500 mL round bottom flask. Then, an oil bath was set to 150 °C and the round bottom flash was placed in the oil bath with a magnetic stirring rod to ensure an even charring. After 30 minutes, the eggshells were removed, and the preparation of uncharred eggshells follows.

C. Preparation of Teabags:

- **i.** Bemliese teabags were submerged into iron oxide nanoparticles dissolved in H_2O for 1 h without stirring. After which, they were hung and left to dry for 24 h in a cool environment. 5 $g \cdot L^{-1}$ of uncharred and mechanically grinded eggshells was added into the teabag, and the top was tied off with locally sourced 100% cotton string.

D. Apparatus:

- **i.** A 1800 UV-visible spectrometer (Shimadzu, Canby, OR, USA) was used with 7 mm quartz cuvettes for the absorbance measurements.
- ii. A FEI Helios Nanolab 660 FIB-SEM was used for scanning electron microscopy (SEM) imaging and energy-dispersive X-ray spectroscopy (EDX) analysis to characterize teabag fibers, nanoparticle embedding, and arsenic distribution.
- **iii.** ATR-FTIR Spectroscopy was performing using a Fourier transform infrared (FTIR) spectrometer (Thermo Scientific Nicolet Summit X) equipped with an Everest attenuated total reflectance (ATR) accessory was used for infrared spectral analysis. Spectra were collected over the range 400–4000 cm^{-1} using 128 scans at a spectral resolution of 16 cm^{-1}**.**

E. Reagents:

- **i.** All chemicals were of analytical grade and purchased from Sigma-Aldrich (Vale Road, Arklow, Co. Wicklow, Ireland), unless otherwise stated. Sodium meta-arsenite ($NaAsO_2$), (Fisher Scientific, Leicestershire, UK), were used to prepare stock solutions at concentration 1000 $\mu g \cdot mL^{-1}$ in DI H_2O. Working standards were prepared by serial dilutions. Potassium iodate (KIO_3), Leucomalachite Green dye ($C_6H_5CH[C_6H_4N(CH_3)_2]_2$), and sodium triacetate trihydrate ($C_2H_3NaO_2 \cdot 3H_2O$) were prepared by weighing

out an appropriate amount and dissolving it in DI H_2O. Hydrochloric acid (1M) was used to prepare hydrochloric acid solutions with various concentrations in DI H_2O. DI H_2O was used for dilution of reagents and samples.

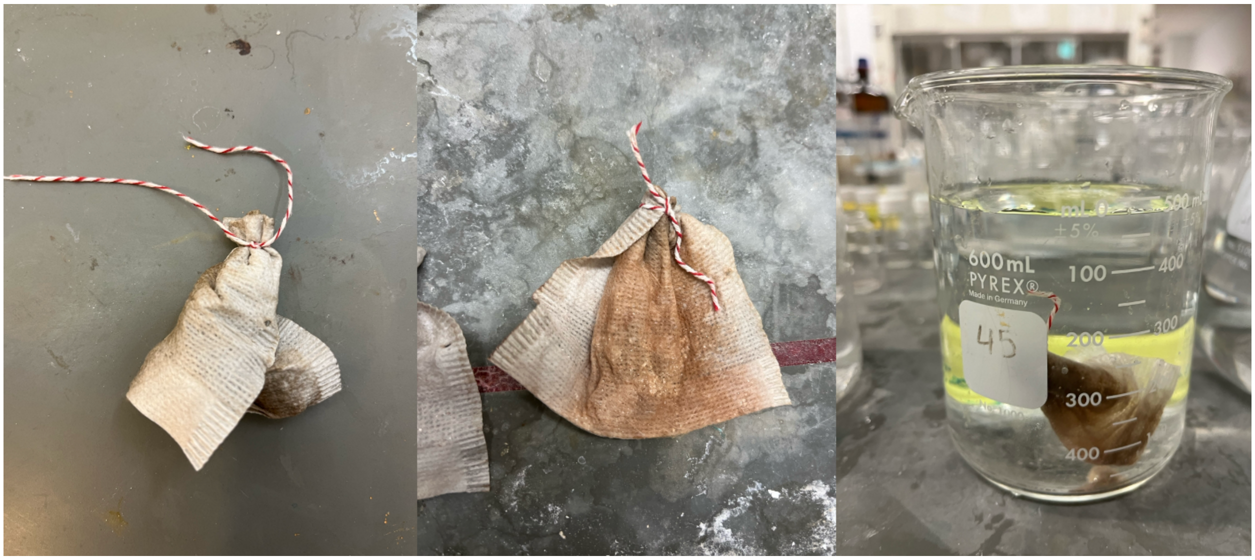

Figure S23. Images of a Teabag embedded with MIO-NP's for 1H without agitation.

6. References

(1) Lace, A.; Ryan, D.; Bowkett, M.; Cleary, J. Arsenic Monitoring in Water by Colorimetry Using an Optimized Leucomalachite Green Method. *Molecules* **2019**, *24* (2), 339.

(2) IARC Working Group on the Evaluation of Carcinogenic Risks to Humans. Arsenic, Metals, Fibres and Dusts. IARC Monographs on the Evaluation of Carcinogenic Risks to Humans, No. 100C; International Agency for Research on Cancer: Lyon, France, **2012**. https://www.ncbi.nlm.nih.gov/books/NBK304380/.

(3) Aziz, S. N.; Aziz, K. M.; Boyle, K. J. Arsenic in Drinking Water in Bangladesh: Factors Affecting Child Health. *Front. Public Health* **2014**, *2*, 57. DOI: 10.3389/fpubh.2014.00057.
